# Prediction of Dissolved Organic Carbon Concentrations in Inland Waters Using Optical Proxies of Aromaticity

**DOI:** 10.1021/acs.est.5c05408

**Published:** 2025-07-30

**Authors:** Kathleen R. Murphy

**Affiliations:** † Department of Architecture and Civil Engineering, Chalmers University of Technology, Gothenburg 41298, Sweden; ‡ Department of Building and Environmental Technology, Lund University, Lund 22363, Sweden

**Keywords:** CDOM, ARIX, ARINT, spectral slope, DOC, monitoring

## Abstract

The chemical structures of dissolved organic compounds in natural waters, including the degree of aromaticity, affect their physical, chemical, and biological properties and ultimately the fate of carbon in aquatic systems and during water treatment. Herein, a new fluorescence-based aromaticity index named ARIX is shown to link the composition of aquatic dissolved organic matter to its aromaticity across diverse aquatic systems in both bulk DOM and extracts. ARIX predicts SUVA, a widely used proxy of aromaticity, more accurately than the prevailing optical indices. It also predicts the percentage of polycyclic aromatic and polyphenolic molecular formulas determined by FT-ICR MS and the ratio of “humic substances” to “building blocks” fractions determined by LC-OCD, indicating that it is additionally a proxy of DOM molecular weight. In waterbodies exhibiting decoupling between DOC and absorbance linked to biogeochemical processing, DOC concentrations are more accurately predicted by using a multilinear model to account for interactions between light absorption and aromaticity. The results deliver new insights into widely discussed trends in DOM optical properties and the molecular structures underlying optical measurements in the aquatic milieu. They further represent an important step toward improved real-time monitoring of DOC concentration, reactivity, and fate.

## Graphical abstract

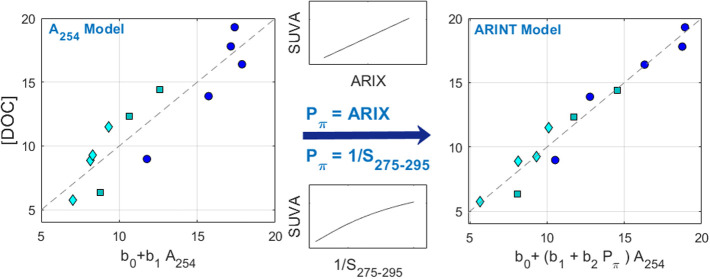

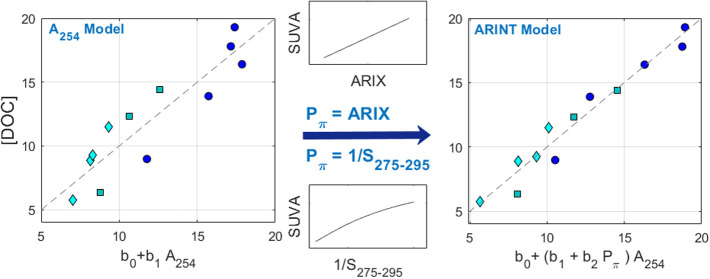

## 1 Introduction

Predicting the fate of dissolved organic matter (DOM) in aquatic systems requires the ability to detect changes in the chemical composition of DOM.^1,2^ DOM consists of potentially millions of compounds of varying age and structural complexity, including those derived from the degradation of biomass, as well as compounds released as byproducts of metabolism or chemical processes.^3^ In river systems the molecular characteristics of DOM affect ecosystem health^4^ and determine whether DOM will leave the water column via biological or photochemical mineralization, flocculation/precipitation, or adsorption, or be transported downstream and stored in the deep sea.^2,5–8^

DOM aromaticity is widely studied due to its influence on wide-ranging chemical and biological processes in water. Many aromatic compounds resist degradation due to their stable conjugated π-electron systems; when combined with the continuous export of aromatic DOM from land, this contributes to the overall carbon storage of aquatic systems over long time scales.^1,9^ Aromatic compounds control primary production by attenuating light underwater and by binding and retaining nutrients within their molecular structures.^10,11^ During drinking water treatment, large polyaromatic compounds are more susceptible to flocculation^12,13^ and compete more effectively with micropollutants for sites on adsorption filters, leading to their premature saturation.^14,15^ The selective removal of polyphenolic and other compounds during water treatment further affects the abundance, types, and toxicity of disinfection byproducts formed during subsequent reactions with chlorine.^16^

DOM aromaticity, referring to the proportion of carbon atoms associated with aromatic bonds, is a bulk property of the pool of molecules that comprise DOM.^17^ Percent aromaticity determined using carbon-13 nuclear magnetic resonance, ^13^C NMR, is strongly correlated to the ratio of UV absorption at 254 nm normalized to DOC concentration, termed specific UV absorbance or commonly SUVA.^17,18^ In natural waters containing low concentrations of dissolved iron, SUVA typically spans the range 1–6 m^2^ g_C_^–1^.^19,20^ Although SUVA is routinely measured as a proxy of aromaticity, it has technical shortcomings due to requiring two different instruments (a carbon analyzer and a spectrophotometer), which starkly increases measurement costs and negatively affects immediacy, accuracy, and precision.^19,21^

Fluorescence spectroscopy is widely used to study DOM composition and two fluorescence indices based on simple emission ratios have been proposed as proxies of aromaticity:^18^ the “fluorescence index” (FI or FIX), and the “humification index” HIX.^22,23^ Also, the “biological” and “freshness” indices (“BIX” and “β/α”), although originally proposed as proxies of autochthonous DOM, often correlate with SUVA.^18,24,25^ However, all such indices have significant drawbacks when predicting aromaticity due to low sensitivity and/or nonlinear responses.^18,19^ Also, the relationship between index values in DOM extracts versus bulk DOM is unclear as is the theoretical basis underpinning their selection; furthermore, demonstrated links to specific DOM molecular structures are missing.^18,26^

Recently, a multispectral fluorescence index (“PARIX”) derived using parallel factor analysis (PARAFAC) was shown to predict SUVA more accurately than FIX, BIX, and HIX in a cross-continental model that included samples from Europe, North America, Africa, and Asia. PARIX also explained differences in DOC removal between French rivers with different SUVA subjected to several standardized treatments.^19^ PARIX in that study was defined as the ratio of two PARAFAC components, one with peak emissions above 500 nm at excitation wavelengths below ∼450 nm and the other with peak emissions near 400 nm at excitations below ∼350 nm. However, while often correlating with water quality parameters,^27^ PARAFAC ratios are usually considered to be site-specific with limited transferability to new contexts.^18^ Also, the requirement to perform a PARAFAC analysis in order to obtain PARIX has practical limitations in monitoring applications, since PARAFAC requires many different samples and a relatively complicated data processing procedure that has yet to be successfully automated.^28^

The present study aimed to provide a robust fluorescence index for predicting DOC aromaticity in bulk DOM and in DOM extracts by drawing upon insights obtained from PARAFAC modeling. It further aimed to link the new fluorescence index to molecular compositions determined by Fourier transform ion cyclotron resonance mass spectrometry (FT-ICR-MS) and DOC fractions measured by size-exclusion liquid chromatography with organic carbon detection (LC-OCD). Finally, it was aimed to improve the estimation of DOC concentrations from optical measurements by accounting for the interaction between light absorption and aromaticity, with aromaticity represented by proxies derived from fluorescence and absorbance spectroscopy. The work was performed by reanalyzing nine published data sets spanning the continents and the river-to-ocean continuum. The results provide new insights into the molecular structures underpinning optical measurements and a technical basis for real-time *in situ* monitoring of DOC aromaticity and concentration in inland waters.

## 2 Materials and Methods

### 2.1 Data Sets

Nine published data sets (*N* = 1340) were reanalyzed in this study (Table 1 and Supporting Information Table S1). The data sets were created by eight independent research groups during the past two decades. They include samples from all seven continents, represent bulk DOM and extracted DOM obtained using three isolation techniques, and span inland surface waters (rivers, lakes, drinking water plants), groundwater, coastal waters, and the ocean. At a minimum, each data set contained SUVA measurements plus fully-corrected fluorescence excitation–emission matrices (EEMs). SUVA was determined according to the traditional USEPA method, which divides absorbance at 254 nm measured on a spectrophotometer (*A*_254_) by DOC concentration measured on a separate TOC analyzer,^21^ or was measured by LC-OCD, which combines both detectors in a single instrument,^29^ after bypassing the chromatographic column. In data sets where both SUVA and DOC were available from the LC-OCD (*SUEZ*, *S. America*), *A*_254_ was calculated as SUVA_LC_/DOC_LC_. In all other cases, *A*_254_ was measured using a dedicated spectrophotometer. Spectral absorbance measurements were additionally available for all data sets except *SUEZ*.

**Table 1:** List of Studied Data Sets^a^

data set	*N*	site description	refs
*Alaska Rivers*	53	Boreal North America: rivers	^32^
*Yukon Lakes*	90	Boreal North America: lakes	^33^
*Everglades*	12	Subtropical North America: rivers	^34^
*SUEZ*	58	Europe, USA, Mediterranean, Cameroon: lakes, rivers and water treatment plants	^19^
*Horsens*	325	Danish river and tributaries	^35^
*Australia*	120	Australian river and tributaries	^36^
*Congo*	135	African river and tributaries	^37^
*S. America*	106	South American headwater streams in Brazil, Chile, and Uruguay	^38^
*Isolates*	37	North America, Europe, and Antarctica: lakes, rivers, estuaries, marine	^20^

a All data sets contain optical measurements performed on bulk DOM, except for *Isolates*, which contain measurements performed on DOM extracts.

Fluorescence intensities were measured on filtered samples in a 1 cm cell using a scanning excitation–emission (EEM) fluorometer. Absorbance was measured in a 1 cm cell within an *Aqualog* fluorometer or else using a dedicated UV–vis spectrophotometer with a 1, 5, or 10 cm cell (Table S1). In this article, SUVA is expressed in units of m^2^ g_C_^–1^ (“meters squared per gram of carbon”), which is a simplification of (i.e., equivalent to) the unit L mg C^–1^ m^–1^ (“liters per milligram of carbon per meter”).^30^

In all data sets except for *Isolates*, spectroscopic measurements were performed on bulk water samples. In the *Isolates* data set, measurements were made after first extracting and concentrating the DOM according to standard methods for measuring hydrophobic organic acids (HPOA, *n* = 22), fulvic acids (FA, *n* = 13), or natural organic matter (NOM, *n* = 2).^20^ The *Isolates* data set included relative abundances of several compound classes derived from molecular formulas identified using FTICR-MS following electrospray (ESI) ionization. Kellerman defined the compound classes using a modified aromaticity index, AI_mod_, which indicates the degree of saturation of molecular formulas.^31^ Specifically, polycyclic aromatic compounds were defined as having AI_mod_ above 0.66 and polyphenolic compounds were defined as having AI_mod_ between 0.5 and 0.66 ^20^

### 2.2 Optical Proxies of DOM Aromaticity

Two data sets (*Isolates* and *Everglades*) were used to test the generality of PARIX, i.e., the PARAFAC-based index developed for estimating aromaticity in bulk DOM.^19^ Philibert et al.^19^ defined PARIX as the ratio of two components, with *H*_ii_ representing a long-wavelength component with peak emissions above 500 nm and *H*_iii_ representing a shorter-wavelength component with peak emissions near 400 nm. Specifically, it was tested whether PARIX derived from different PARAFAC models created by different research groups predicts SUVA in bulk EEMs (*Everglades*) or DOM extracts (*Isolates*). In both cases, the raw data consisted of PARAFAC loadings reported in published tables, and PARIX was calculated as the ratio between reported *F*_max_ values for components similar to *H*_ii_ and *H*_iii_. The *Isolates* data set was further used to test whether PARIX predicts the relative abundance of molecular formulas associated with polycyclic aromatic and polyphenolic compound classes.

The *Isolates* data set, as well as the eight data sets comprising whole-water DOM *(Alaska Rivers*.,^32^ *Australia*,^36^ *Congo*,^37^ *Everglades*,^34^ *Horsens*,^35^ *S. America*,^38^ *SUEZ*,^19^ *Yukon Lakes* ^33^), were used to assess correlations between SUVA and a newly identified fluorescence ratio called ARIX. ARIX is defined as the ratio of emission intensities detected at two fixed emission wavelengths (520/390 nm) when excited by light at 320 nm. ARIX tracks the ratio of PARAFAC components identified by Philibert et al.^19^ and referred to as *H*_ii_ and *H*_iii_. Since *H*_iii_ overlaps spectrally with several ubiquitous PARAFAC components having emission peaks between 400–450 nm,^19,39–41^ the ARIX algorithm tracks PARIX using wavelengths on the shoulders of the underlying PARAFAC components instead of the positions of *F*_max_. This is so that ARIX will (to the furthest foreseeable extent) avoid interfering fluorescence, both from overlapping nontarget fluorophores and from Raman scatter.^42^

The slope of the absorbance spectrum measured between 275 and 295 nm (*S*_275–295_) is often used to trace terrestrial DOC in the ocean^43^ and correlates inversely with DOM molecular size and absorptivity.^18,44^ *S*_275–295_ was calculated according to Helms et al.^45^ for use in ARINT models and Yan et al.^44^ for use in DOC_UV_ and DOC_LS_ models. It was not possible to determine *S*_275–295_ for the SUEZ data set because of the lack of absorbance spectra.

### 2.3 Regression Models

For each data set, individual regression models were calculated in MATLAB (ver. 2022a) using the *fitlm* function to predict SUVA with model ŷ = β_1_ (P)­ARIX + β_0_. Models were made with and without MATLAB’s robust statistics option that performs automatic outlier exclusion. Regressions were additionally calculated using four widely used fluorescence indices as the independent variable in place of ARIX. These were FI (“fluorescence index”), HIX (“humification index”), β/α (“freshness index”), and BIX (“biological index”). FI was calculated as the ratio of emission intensities detected at 470 and 520 nm upon excitation at 370 nm.^22^ HIX was calculated according to two different algorithms; HIX is the sum of emissions at 435–480 nm divided by the sum of emissions from 300–345 and 435–480 nm following excitation at 254 nm,^23^ whereas its predecessor HIX_1999_ has the same numerator but the denominator integrates emissions from 300–345 nm only.^46^ BIX was calculated as the ratio of emission intensities detected at 430 and 380 nm upon excitation at 310 nm.^24^ β/α was calculated as the ratio of emission detected at 380 nm to the maximum emission detected at 420–435 nm upon excitation at 310 nm.^25^ The algorithms for BIX and β/α produced very similar regression results, so BIX alone is plotted, although regression statistics for both indices are reported in tables. Similarly, HIX and HIX_1999_ are reported in tables but only HIX is plotted.

To derive a global model linking SUVA with ARIX in whole-water samples, a geometric regression (model II regression) was calculated using *lsqfitgm* code from MBARI with SUVA as the *Y*-variable and ARIX as the *X*-variable.^47^ In contrast to traditional (model I) regression, where *X* is the error-free independent variable and *Y* depends on *X*, model II regressions are used to fit relationships between *X* and *Y* when both contain errors and depend upon a third (unmeasured) variable. This is done by minimizing offsets along both axes equally instead of only along the *y*-axis.^47^ Since seven different fluorometers were used to measure the global data set, the model II regression was most appropriate. Model I regressions were used for individual data sets since each was measured using a single fluorometer.

Prior to regression analyses, outliers were excluded from four data sets. In the *Horsens* data set, ARIX values varied randomly in estuarine samples, indicating a complete loss of measurement sensitivity; thus, all estuarine samples were removed. Conversely, in the *Isolates* data set, where measurements were performed on DOM concentrates, marine samples were retained from Penobscot Bay, the Gulf of Maine, and the Pacific Ocean. In the *Australia* data set, one clearly erroneous sample was excluded, while in *Yukon Lakes*, one sample with extremely high DOC (>120 mg/L) was excluded. In the *Horsens* data set, two samples with unrealistically high SUVA above 7 m^2^ g_C_^–1^ were excluded. Additionally, in 13 riverine samples, SUVA was half the value predicted by ARIX, although ARIX values were consistent with neighboring sites and with measurements from the same site during other sampling campaigns. For these samples, absorbance measurements were 2× higher than expected, which suggests an oversight when recording the path length (5 cm vs 10 cm). Deleting all river samples with SUVA below 2 removed nine such outliers; four others were retained.

### 2.4 Sensitivity Analysis

Fluorescence, absorbance, and DOC measurements have different inherent sensitivities, and instruments from different manufacturers (and even different versions of the same model) have varying levels of sensitivity and bias. A simulation was performed to estimate how much of the scatter in the relationship between SUVA and ARIX in bulk EEMs might be attributable to measurement error. The eight bulk EEM data sets containing 876 samples from inland waters were used to generate simulated data sets. Initially, an “error-free” simulated data set was created with DOC, SUVA, and ARIX chosen to be identical to their values in the real data set, whereas *A*_254_ was recalculated so that the data aligned exactly with the regression equation. Thus, *A*_254_ was obtained by multiplying the equation for predicting SUVA from ARIX by DOC. The resulting data set had a similar distribution of ARIX and SUVA as the original data set but no deviation from the regression line. Thereafter, 100 simulation runs were performed. In each run, an error residual was added to each measured variable (DOC, ARIX and *A*_254_), with this residual selected randomly from an error distribution assumed for the specific type of measurement. In each case, errors were assumed to follow a normal distribution with a mean of zero and standard deviations chosen to reflect typical measurement errors reported for a range of laboratories and instruments (Table S3).^21,48^ The median RMSE value across all 100 simulations estimates how much prediction error in the global model could feasibly be attributed to random measurement error.

### 2.5 DOC Predictions from Optical Measurements

DOC was predicted from optical measurements using linear and nonlinear models. An Aromaticity Interaction (ARINT) Model was developed using a multiple linear regression to predict DOC from *A*_254_, allowing an interaction between *A*_254_ and DOM aromaticity. Aromaticity was represented by an optical proxy, either ARIX or 1/*S*_275–295_, since *S*_275–295_ is inversely correlated to aromaticity.^44^ While it is meaningless to predict DOC from SUVA, models were also investigated with SUVA standing in for an optical proxy to indicate the expected performance of a model in which *A*_254_ interacts with a perfect proxy of SUVA. Multiple linear regressions for ARINT models were performed using the *regress* function in MATLAB with *A*_254_ as the independent variable and DOC as the dependent variable, allowing an interaction between *A*_254_ and one of the above three proxies. Multiple linear regression models typically include all terms with significant interactions as main effects; however, an exception is made for nested variables if including them as a main effect could lead to them taking on meaningless values. Since ARIX and *S*_275–295_ are undefined when absorbance is zero, aromaticity is included as an interaction term but not a main effect.

For comparison with the ARINT models, DOC was additionally predicted according to two recent empirical models derived from nonlinearly transformed absorbance measurements. The “Pan-Arctic” model of Gonçalves-Araujo and colleagues^49^ predicts DOC from CDOM absorption at 350 nm (*a*_350_ m^–1^) and *S*_275–295_ after estimating parameters *C* and *M* in the equation log_10_(DOC/*a*_350_) = *C* + (*M* × *S*_275–295_). The algorithms of Yan and colleagues^44^ predict DOC from CDOM absorption at 275 nm (*a*_275_ m^–1^) combined with spectral slopes obtained in the 275–295 and 380–443 nm range, using the formula DOC = Φ*a*_275_(*S*_275–295_ + 0.078*S*_380–443_ – 0.0084) + DOC_cor_. Using their global DOC_UV_ model, Φ and DOC_cor_ have fixed values of 1507 m·nm·μmolL^–1^ and 32.2 μmolL^–1^, respectively.^44^ Using their local DOC_LS_ model, optimal Φ and DOC_cor_ values are calculated for any specific data set using least-squares fitting, which should produce more accurate DOC predictions than the global model. In the current study, MATLAB (v2023b) was used to obtain optimal Φ and *DOC*_*cor*_ for each data set, and fits were calculated for both the DOC_UV_ and DOC_LS_ models.

Multimodel inference was used to compare the goodness of fit of the above six competing models by balancing fit (determined as −2 × log likelihood) with parsimony, whereby each estimated parameter incurs a penalty.^50^ Ignoring the fit, the DOC_UV_ model is most parsimonious since no parameters are estimated. In comparison, two parameters are estimated when predicting DOC from A_254_ (a slope coefficient and an intercept), when using the Pan-Arctic model (C and M), or when using the DOC_LS_ model (Φ and DOC_cor_). ARINT models draw the largest penalties since three components are estimated, i.e., two regression coefficients and a y intercept. Akaike’s Information Criteria (AIC, AICc, and CAIC) and the Bayesian Information Criterion (BIC) were each calculated in MATLAB. These metrics each calculate slightly different penalties to log likelihood fits based on the number of estimated parameters and (in the case of BIC, AICc, and IC) the number of samples. The model achieving the lowest value for most or all information criteria is preferred according to the dual criteria of fit and parsimony.

## 3 Results and Discussion

### 3.1 Predicting DOC Composition from Fluorescence Ratios

PARIX was an unbiased predictor of the proportion of polycyclic aromatic (PA) and polyphenolic (PP) structures in DOM extracts comprising the global *Isolate* data set, according to molecular formulas measured using FT-ICR MS (Figure 1A). In Figure 1, as in subsequent figures, the dashed lines on either side of the regression line represent 95% confidence bounds for the predicted regression equation. Data are from Table 1 in Kellerman et al.,^20^ representing diverse freshwater and marine samples, with PARIX calculated as the ratio of tabulated scores for PARAFAC component C3 divided by C2. Pacific samples from 21 and 240 m depths conformed to the regression, whereas a deep ocean sample and a river sample diverged (Figure 1A).

**Figure 1:**
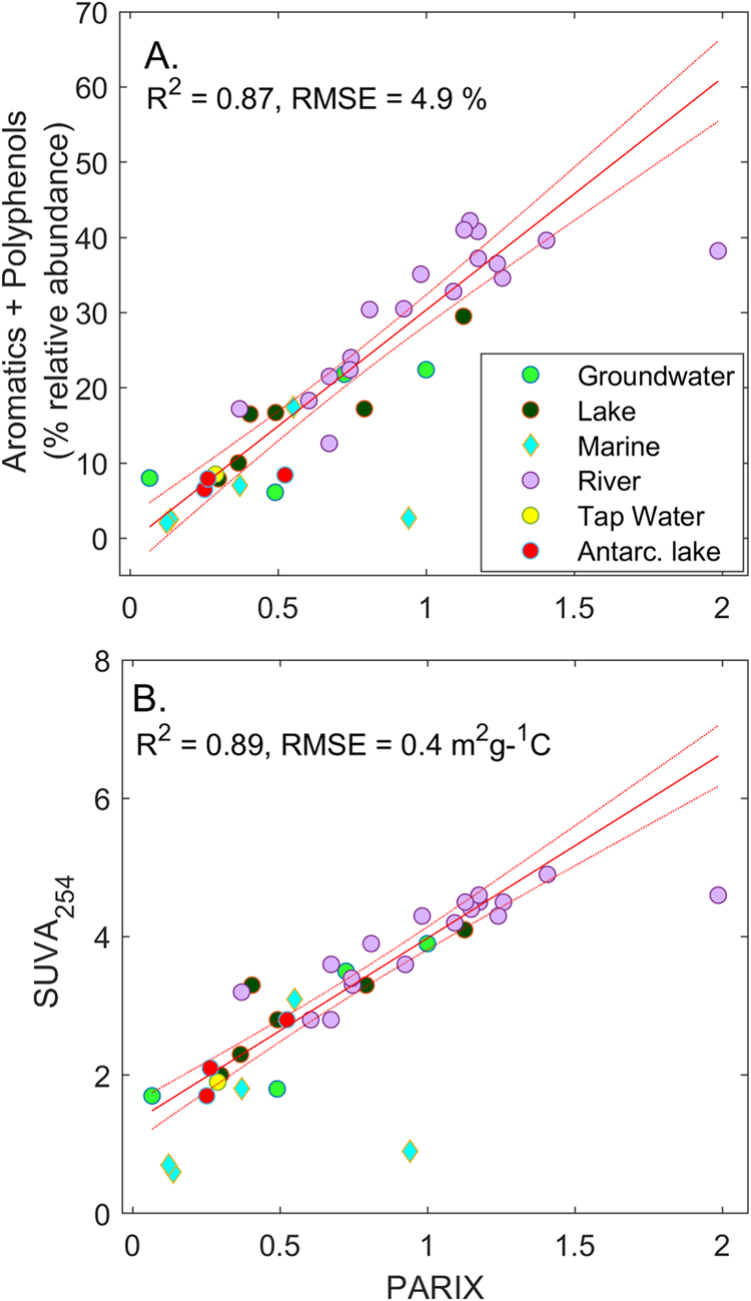
Prediction of DOC aromaticity from PARIX in the global *Isolates* data set. Aromaticity was determined by (A) FTICR-MS; or (B) SUVA. PARIX was calculated from Table 1 in Kellerman et al.^17^ as C4/C3. SUVA has units m^2^ g_C_^–1^, and PARIX is dimensionless.

Equation 1 in Figure 1A estimates the relative abundance of polyphenolic compounds within 5% for samples in which these formulas comprised 2–45% of total formulas.

1 %(PA+PP)=30.28PARIX

Equation 1 has no *y* intercept, indicating that the PARIX denominator will be zero when there are no PA or PP structures. This further implies that all electrospray-ionized molecular formulas identified as polycyclic aromatic and polyphenolic structures in the *Isolates* data set were fluorescent. Whether or not this finding is generalizable to all FT-ICR-MS data sets should be confirmed by future studies. It is especially of interest to test different DOM extraction methods (e.g., PPL), since compounds vary in their affinities to extraction sorbents, and other ionization techniques (e.g., APCI and MALDI), since compounds additionally vary with respect to the efficiency with which they are ionized using different techniques.^51,52^

PARIX also predicted SUVA in samples from inland waters and the coastal ocean (Gulf of Maine, Penobscot Bay), but not in the samples from the central Pacific (Figure 1B). It is likely that the carbon in Pacific ocean samples was extensively photobleached during transport from land to open ocean, causing a decoupling between carbon content and color.^30^ The same river sample was again an outlier, indicating PARIX to be responsible for the divergence.

PARIX accurately predicted SUVA in bulk EEMs from two brackish river systems in the Florida *Everglades* (Figure 2A). PARIX in Figure 2A is calculated from Table 2 in Timko et al.^34^ as the ratio of scores for PARAFAC component C4 divided by C5. This reveals a tight correlation between PARIX and SUVA spanning both river systems (*R* ^2^ = 0.96). These two data sets demonstrate linearity between PARIX and two different proxies of DOC aromaticity. Importantly, these relationships hold for both bulk DOM and DOM extracts, and are independent of the specific PARAFAC model used to calculate PARIX.

**Figure 2:**
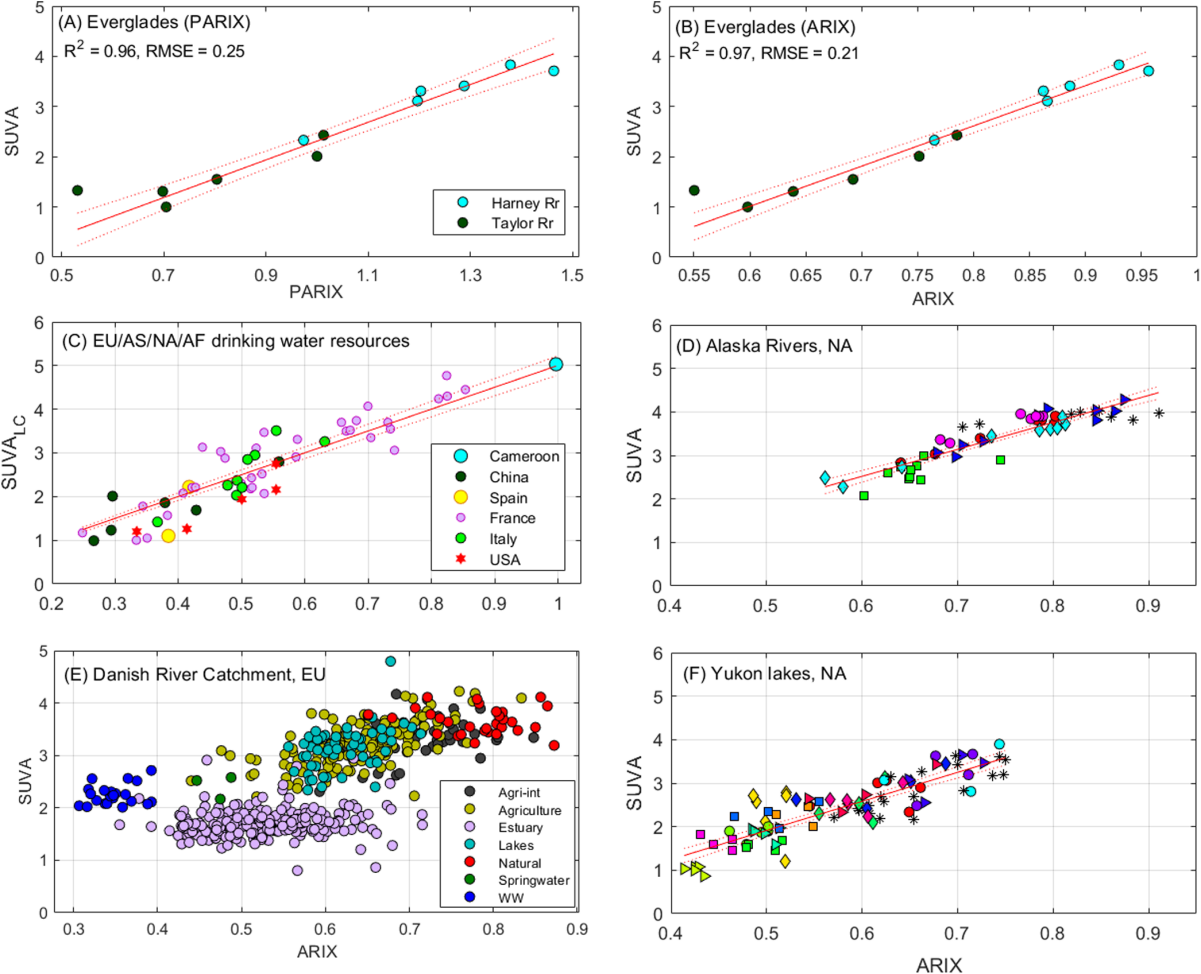
Prediction of SUVA from (P)­ARIX in bulk surface waters. (A) PARIX; (B–F) ARIX. Samples are from inland waters and water treatment plants in Europe (EU), North America (NA), Asia (AS), and Africa (AF).^12,29,31–33^ In A, B, D, and F, each new symbol represents a different river or lake. SUVA has units m^2^ g_C_^–1^ and ARIX is dimensionless.

In the diverse treated and untreated water samples comprising the *SUEZ* data set, ARIX was an unbiased predictor of the ratio of humic substance (HS) to building block (BB) fractions determined by LC-OCD (Figure 3 and eq 2). While HS is understood to comprise high molecular weight humic substances, the BB fraction represents lower molecular weight weathering and oxidation products of humic substances.^29^

2 $$\frac{\mathrm{HS}}{\mathrm{BB}}=6.1\mathrm{ARIX}$$

**Figure 3:**
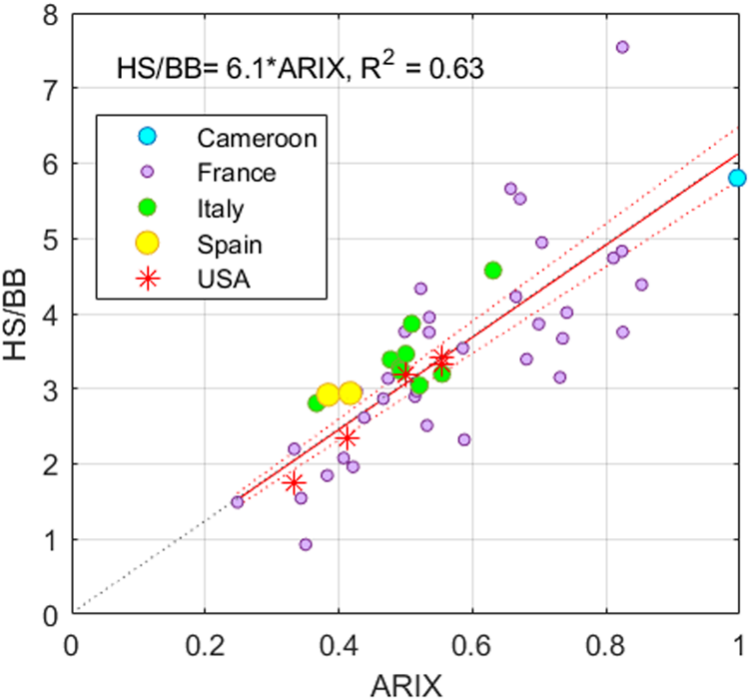
Prediction of the LC-OCD composition from ARIX. Samples represent treated and untreated surface and groundwaters from the *SUEZ* data set (HS: humic substances, BB: building blocks). Both axes represent dimensionless parameters.

The simple relationships in eqs 1–2 are notable considering that FT-ICR MS and LC-OCD characterize a wide range of both colored and uncolored molecular structures. Both techniques also have different inherent biases. In FT-ICR MS analysis, electron spray ionization leads to variable ionization of different molecular formulas, affecting the relationship between signal strength and concentration. In LC-OCD analysis, chromatograms achieve incomplete separation of HS and BB signals, so must be combined with chromatographic deconvolution,^29^ similar to using PARAFAC to deconvolute overlapping fluorescence signals when calculating PARIX. The linear correlation between a fluorescence ratio and LC-OCD ratio in eq 2 provides support for both deconvolution techniques. However, it is important to recognize that regression slopes could vary between data sets depending on instrument resolution, ionization source, spectral biases, and the specific algorithms used to resolve overlapping peaks. Also, Figure 3 shows that ARIX predicts DOM molecular weight in addition to aromaticity, confirming earlier observations about the interdependence of these properties.^18^

Whether aromatic molecules in the DOC pool exhibit fluorescence depends on their specific structures and electronic properties. The ARIX numerator tracks a long-wavelength fluorescence component identified repeatedly in PARAFAC analyses^20,53^ and usually attributed to extensively π-conjugated polyaromatic structures.^54^ The denominator tracks a short-wavelength component with a secondary excitation maximum around 330 nm and emission peak below 400 nm.^19,41^ This is similar to several oxidized fluorophores consisting of a single aromatic ring with attached carboxy, hydroxy, and methoxy groups, e.g., vanillic acid, syringic acid, and acetovanillone^55^ albeit with longer absorption, possibly indicating additional substitution and/or the presence of a short, conjugated side chain, as in ferulic acid or coniferyl alcohol, or a conjugated heterocycle, as in coumarin.^5,55,56^

### 3.2 Predicting SUVA from (P)­ARIX in Inland Waters

In individual data sets, ARIX and PARIX were reliable predictors of SUVA by linear regression (Figure 2 and Table S2). In the *Everglades* data set representing two river systems draining a tropical wetland, ARIX predicted SUVA more accurately than PARIX with RMSE = 0.21 m^2^ g_C_^–1^. Low prediction errors (0.24–0.36 m^2^ g_C_^–1^) were also observed for two high-latitude data sets consisting of six rivers (Figure 2d) and 15 hydrologically isolated lakes (Figure 2f) in the Yukon basin, Alaska.^32,33^ In most data sets, ARIX and/or PARIX outperformed traditional fluorescence indices when predicting SUVA. Average prediction errors (m^2^ g_C_^–1^) in increasing order for whole-water data sets were: ARIX (0.35) < β/α (0.43) = BIX (0.43) < HIX_1999_ (0.52) < HIX (0.55) < FI (0.58) (Table S2 and Figures S1–S4). Thus, although FI and HIX are the two fluorescence indices used most frequently to predict DOM aromaticity,^18^ both were significantly poorer predictors of SUVA than ARIX and the two “biological” indices.

ARIX correlated with SUVA in marine samples from the *Isolates* data set, but there was no correlation between ARIX and SUVA in the *Horsens* estuary (Figure 2e). Horsens river flows past relatively pristine sites in its upper reaches, then through an agriculturally impacted landscape, and ultimately past a wastewater treatment plant near the entrance to the estuary.^35^ This progression is seen by decreasing the SUVA and ARIX while moving downstream. Estuary sites featured high salinities (32 ppt) and low SUVA (1.6–2.4 m^2^ g_C_^–1^) as is typical for marine samples,^30^ and at these sites ARIX varied randomly. It is likely that for *Horsens* in contrast to *Isolates*, the rapid dilution of terrestrial DOM in the estuary caused fluorescence intensities to drop below detection limits for quantifying ARIX. This highlights the need to establish detection limits for using ARIX to predict DOC in aquatic systems that have low DOC concentrations or significant seawater intrusion.

Variations in water chemistry affect the prediction of DOC aromaticity from fluorescence ratios. Changes in pH from 4 to 8 have small systematic effects on fluorescence ratios HIX, BIX, and FIX,^57^ but pH effects on ARIX have not been examined. Assuming a significant effect of pH, this could manifest as a lower coefficient of determination (*R* ^2^) when pH varies within the data set. Fe­(II) and Fe­(III) cations interfere with SUVA due to light absorption by aqueous iron complexes,^58^ and both species, as well as several other metals (e.g., Cu, Hg, Al), reduce fluorescence via quenching reactions.^59^ In the presence of quenching metals, nonlinearities would be expected to arise between SUVA and ARIX because A_254_ increases with increasing iron concentrations, whereas ARIX will decrease due to the preferential quenching of long-wavelength fluorescence.^60–62^

Overall, the regression slope terms (β_1_) for predicting SUVA from ARIX varied between data sets, with the North American data sets having steeper slope terms than their European, Australian, and African counterparts (Table S2 and Figures S1–S3). Comparing regressions equations for pairs of data sets, β_1_ was not statistically different in *Alaska Rivers* (6.36 ± 0.41) vs *Yukon Lakes* (7.03 ± 0.45) or *SUEZ* (5.8 ± 0.36), although the latter data set is dominated by European samples. Also, β_1_ for *Yukon Lakes* was not statistically different from *Everglades* (8.01 ± 0.48). The remaining four data sets had significantly lower β_1_: *S. America* (4.51 ± 0.58), *Congo* (3.71 ± 0.50), *Horsens* (Denmark) (3.26 ± 0.16), and *Australia* (2.08 ± 0.19). Differences in slope can arise from compositional variation between DOM in different data sets linked to differences in source or biogeochemical processing. A small slope indicates that either the higher molecular weight fraction producing long-wavelength fluorescence is less efficient at emitting light than the same fraction in a data set with a larger slope, or that the lower molecular weight fraction producing short-wavelength fluorescence is relatively more efficient.

### 3.3 Global Models for Predicting DOM Aromaticity

The *SUEZ* and *Isolates* data sets each span several continents and multiple biomes yet produced similar or higher *R* ^2^ than several geographically restricted data sets. For the *Isolate* data set, fluorescence measurements were performed on extracted DOM, which probably limited interfering matrix effects and improved signal/noise, especially for the marine samples. However, the SUEZ data set was measured on whole-water EEMs and still indicates a single regression for predicting SUVA from ARIX regardless of sample origin.

Plotting all eight whole-water data sets together (Figure 4) showed ARIX to be confined within the range 0.15–1.1, with most samples falling between 0.25 and 0.9. Excluding *Everglades*, all data sets were reasonably well captured by a single regression equation. Equation 3 is derived from a geometric (model II) regression^63^ and has slope 6.07 ± 0.14 and intercept −0.67 ± 0.09.

3 SUVA=6.1ARIX−0.7

**Figure 4:**
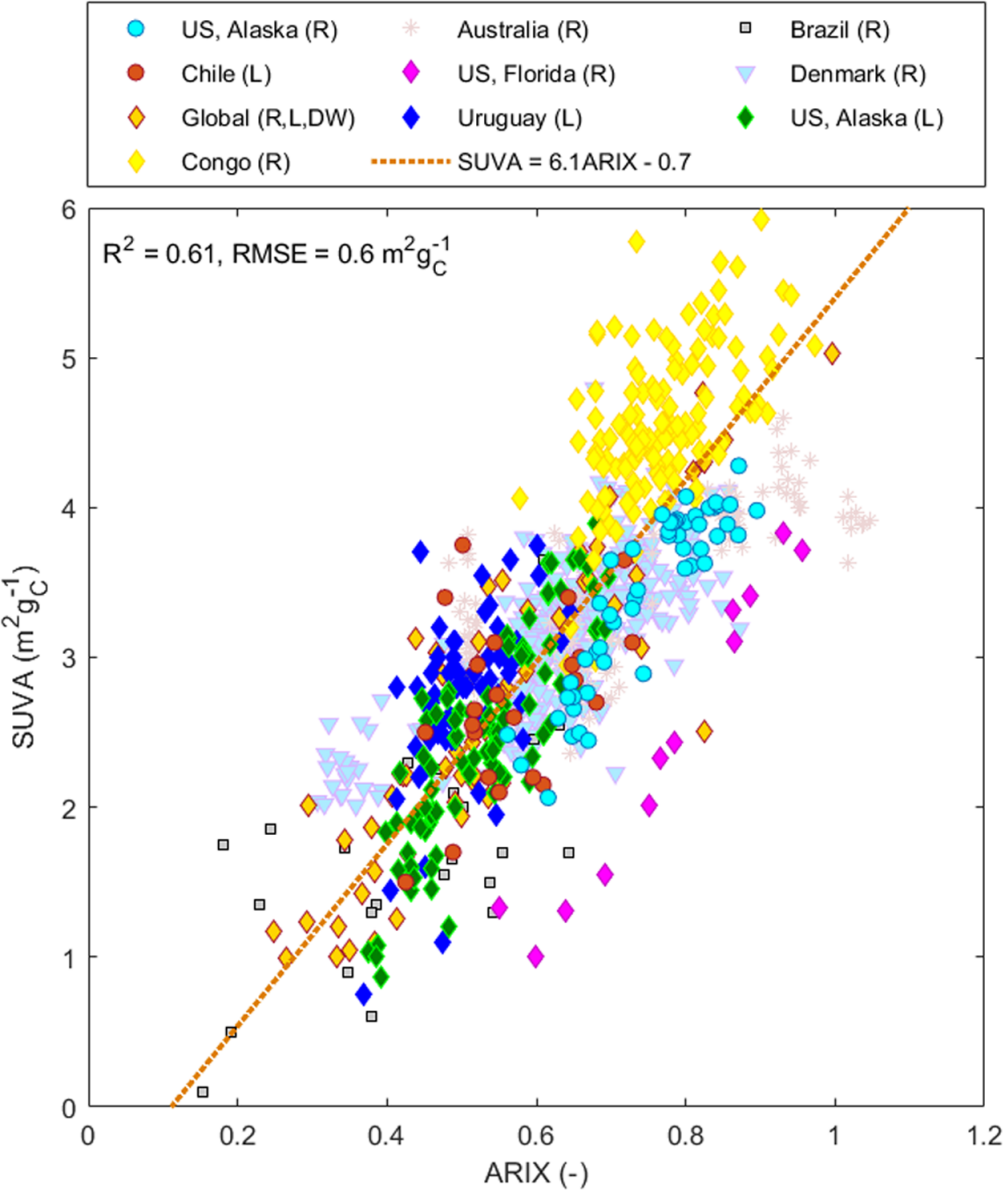
Prediction of SUVA from ARIX in bulk DOM from global surface water and groundwaters. Samples represent fresh and brackish waters (R: rivers, L: lakes, DW: treated and untreated drinking water).

The *Everglades* data set showed a similar slope as the overall trend, except with ARIX transposed right by ∼0.3 units or SUVA transposed down by ∼1 unit. This may well reflect true variation with a source that is presently unknown. It is difficult to explain in terms of instrumental artifacts, since a constant offset in one detector does not produce a constant offset along the SUVA or ARIX axis.

A strong correlation between SUVA and ARIX was also seen for the DOM extracts. For the *Isolates* data set, the robust regression of SUVA upon ARIX indicated a strong correlation (*R* ^2^ = 0.82, RMSE = 0.50) with slope = 4.50 ± 0.10 and no significant intercept (*t* = −1.77, *p* = 0.08, *df* = 35) (SI Figure S3c-iv). A similar slope (4.50 ± 0.09) is obtained after excluding the three Pacific Ocean extracts and using an ordinary linear regression without intercept, producing eq 4 (*R* ^2^ = 0.84, RMSE = 0.40, *df* = 33)

4 SUVA=4.5ARIX

Extending Weishaar’s equation linking ^13^C NMR aromaticity to SUVA in XAD-8 isolates^17^

5 percent aromaticity=6.52SUVA+3.63=29.3ARIX+3.63

Assuming that fluorescence properties of Weishaar’s isolates followed similar trends to the *Isolates* data set, eq 5 suggests that in DOM isolated on XAD resins, percent aromaticity can be roughly estimated as 30 × ARIX.

Whereas diverse molecular compositions probably explain much of the variability in regression slope coefficients among the nine data sets, some variability may reflect artifacts. Comparing the global data set of DOM isolates (eq 4) with bulk DOM (eq 3), a lower slope was obtained for the DOM extracts. A possible explanation is that the molecules responsible for the ARIX numerator and denominator have different selectivity toward extraction.^53,64^ This is difficult to verify due to a lack of sufficiently detailed studies of wavelength-dependent extraction efficiencies for XAD extracts. However, Wünsch et al.^65^ reported that for samples from arctic fjords extracted on PPL sorbents, the longest wavelength PARAFAC component with maximum near 500 nm was extracted with efficiency less than half that of a component with maximum near 410 nm (17 ± 4% for C_500_ vs 50 ± 15% for C_410_). This will produce a smaller slope in the regression of SUVA upon ARIX for PPL isolates compared with bulk DOM.

Among the eight whole-water data sets, artifacts could instead arise from systematic differences in measurement protocols and instruments. For example, the desire to stabilize samples prior to shipping overseas introduced logistical challenges that were solved differently in different studies. *Congo* and *S. America* samples were, in each case, transported to Europe for analysis. Prior to transportation, *Congo* samples were filtered (0.2 μm) and refrigerated, whereas *S. America* samples were filtered, then acidified and frozen, and prior to measurement, were thawed, refiltered, and neutralized with a base. Although some studies have reported that both acidification and freezing effects on DOM optical properties were fully reversible upon subsequent neutralization and/or thawing,^66,67^ others have observed permanent changes in DOM concentration and composition including altered fluorescence intensities and SUVA.^68–70^ Alterations appear to result from changes in the conformation, aggregation, and/or hydrolysis of dissolved molecules and are especially observed in samples with higher DOC concentrations and/or higher aromaticity.

Interlaboratory comparison exercises often highlight biases arising from slightly different procedures and analytical instruments, including for fluorescence spectroscopy,^48^ DOC and SUVA,^21^ and FTIR-MS.^71^ SUVA measured by the USEPA method is the ratio of measurements derived from a spectrophotometer and a carbon analyzer, both with different inherent sensitivities and biases, making it highly susceptible to both random and systematic errors.^21^ In developing the USEPA standardized method for SUVA analysis, Potter and Wimsatt^21^ compared SUVA measured on duplicate samples using five different commercial DOC analysers placed in the same laboratory. Despite these efforts to standardize measurement conditions and the use of a single spectrophotometer to measure absorbance in a 10 cm cell, a high standard deviation (∼0.3 m^2^ g_C_^–1^) was observed across all measurements.

In the current study, since seven different laboratories and 19 different detectors were used to derive eq 3 and Figure 4, systematic biases related to different instruments and measurement protocols are unavoidable. In data sets where SUVA was measured using both the USEPA method and using LC-OCD, it was observed that the strongest correlations were obtained between ARIX and SUVA_LC_. For the *SUEZ* data set, deviations could be traced to the lab spectrophotometer because DOC measured by LC-OCD was identical to DOC measured using the lab carbon analyzer (β_1_ = *R* ^2^ = 1, RMSE = 0.01 mg L^–1^) yet SUVA was 11% lower than SUVA_LC_ (β_1_ = 0.89, *R* ^2^ = 0.85). In two other data sets, traditional SUVA correlated only weakly with SUVA_LC_.

A sensitivity analysis indicated that around a third of the variability in Figure 4 can be explained by purely random measurement errors under realistic assumptions about the precision of fluorescence, absorbance, and DOC detectors (simulated/observed RMSE = 32.4%, Figure S4 and Table S3). The relationship between ARIX and SUVA is especially sensitive to absorbance errors because *A*_254_ is typically measured in a 1 cm cell and then multiplied by 100 to produce SUVA. Individual data sets encompassing geographically diverse samples and precise detectors may therefore provide more realistic estimates of the variability to be expected when predicting SUVA from (P)­ARIX across systems. For the *Isolates* (extract) and *SUEZ* (whole-water) data sets, RMSE was 0.40–0.50 m^2^ g_C_^–1^ (Table S2).

### 3.4 DOC Prediction Models

For nearly all whole-water data sets, an improved correlation between DOC and *A*_254_ was achieved by assuming an Aromaticity Interaction (ARINT) Model. This model extends a simple regression of DOC on *A*_254_ alone (the “base model”) by adding a term to represent the interaction between absorbance and aromaticity

6 $$\begin{array}{l} \mathrm{DOC}={b_{0}+b}_{1}A_{254}+b_{2}{A_{254}P}_{\pi }\\ {(\mathrm{DOC}-b}_{0})=A_{254}\left(b_{1}+b_{2}P_{\pi }\right) \end{array}$$

In eq 6, *P*_π_ represents a proxy of aromaticity, either ARIX, SUVA or 1/*S*_275–295_, while *b*_0_, *b*_1_, and *b*_2_ are coefficients in the multilinear regression. SUVA and *A*_254_ have units m^2^ g^–1^ and m^–1^ respectively, while DOC has unit mg L^–1^, which simplifies to gm^–3^. ARIX has no units, and *S*_275–295_ has units μm^–1^. The coefficient *b*_0_ has the same units as DOC, i.e., gm^–3^, while *b*_1_ has unit gm^–2^. The unit of *b*_2_ depends on the choice of *P*_π_; when *P*_π_ = SUVA, *b*_2_ has unit g^2^ m^–4^, when *P*_π_ = ARIX, *b*_2_ has unit gm^–2^, and when *P*_π_ = 1/*S*_275–295_, *b*_2_ has unit Mg m^–1^.

The *y* intercept *b*_0_ represents uncolored DOC, and thus (DOC – *b*_0_) represents colored DOC, herein termed cDOC. The equation can be further rearranged to predict *A*_254_ from cDOC.

7 $$A_{254}=\frac{\left(\mathrm{DOC}-b_{0}\right)}{\left(b_{1}+b_{2}P_{\pi }\right)}=\frac{c\mathrm{DOC}}{\left(b_{1}+b_{2}P_{\pi }\right)}$$

Equation 7 takes the form of the Beer–Lambert law *A* ∝ εl*c*,^72^ where *c* is the molar concentration, *l* is the path length, and ε is the molar absorptivity. In samples from data sets that conform to eq 7 the molar absorptivity of chromophoric DOC is proportional to 1/(*b*_1_ + *b*_2_ *P*_π_).

The results of using eq 6 with different *P*_π_ proxies to predict DOC in the eight whole-water data sets are provided in Figures S5–S12 and Tables S4–S5. Using *P*_π_ = SUVA indicated how well the model would theoretically have performed had *P*_π_ been a perfect proxy of SUVA. For seven data sets (*Yukon Lakes, Horsens, Alaska Rivers, Everglades, Australia, Congo, SUEZ*), the ARINT model with *P*_π_ = SUVA reduced prediction errors relative to the base model by 43–88%, while for *S. America*, error decreased by 12% (Supporting Information Table S4). In these models, coefficient *b*_1_ was always positive and in the range of 0.16–0.86. Coefficient *b*_2_, which scales the interaction between *A*_254_ and aromaticity, was always negative, indicating that the rate of increase in DOC with increasing *A*_254_ decreased with increasing SUVA. *Congo* had the smallest absolute *b*_2_ (−0.02), whereas *Yukon Lakes* had the largest (−0.19).

Using *P*_π_ = ARIX in eq 6 improved DOC predictions relative to the base model in all data sets except *Congo*, although always by less than *P*_π_ = SUVA models (Supporting Information Table S4). The largest improvements occurred for *Everglades* and *Yukon Lakes* with prediction errors decreasing by 47% and 35%, respectively, relative to the base model (Figure 5 and Table S3). For *SUEZ*, *Horsens*, and *S. America*, prediction errors decreased by 21–26%. Very small but statistically significant improvements were also obtained for *Alaska Rivers* and *Australia* (1–4%) As in the *P*_π_ = SUVA models, in valid *P*_π_ = ARIX models the coefficient *b*_1_ was always positive (0.23–1.0) while the coefficient *b*_2_ was negative (−0.04 to −1.18) (Table S4).

**Figure 5:**
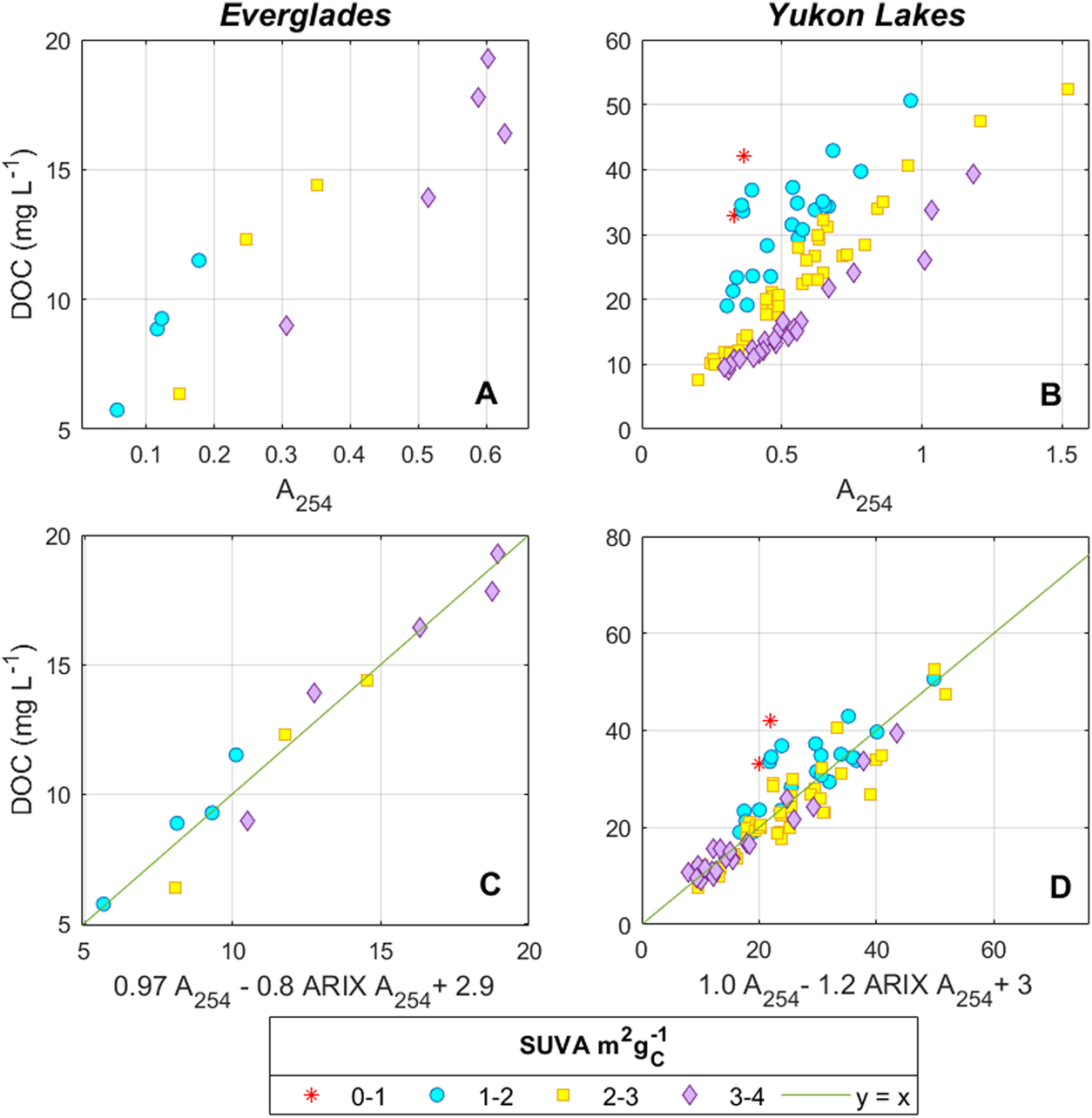
Prediction of DOC concentrations from absorbance and ARIX in hydrologically isolated catchments. Top row: Measured DOC vs A_254_ in (A) *Everglades*; (B) *Yukon Lakes* data sets. Bottom row: Measured DOC vs DOC predicted from eq 6 for (C) *Everglades*; and (D) *Yukon Lakes*.

Using *P*_π_ = 1/*S*_275_295_ led to large improvements in DOC predictions for *Everglades* and *Yukon Lakes* (Table S4), with prediction errors decreasing by 65% and 46% relative to the base model. Modest improvements were obtained for *Alaska Rivers* (9%) and *S. America* (12%), while *Horsens* improved by 1%. Across these data sets, SUVA was nonlinearly correlated with *S*_275_295_ with equation SUVA = 8.48 exp­(−0.060*S*_275_295_) (RMSE = 0.54, *R* ^2^ = 0.57) (Figure S13). No significant improvement was obtained for *Australia* or *Congo*. The slope of the nonlinear relationship between SUVA and *S*_275_295_ changes most slowly when SUVA is small (Figure S14), indicating that *S*_275_295_ will be most sensitive when predicting DOC within the lower range of DOM aromaticities. This would increase its relevance for predicting DOC concentrations in nearshore and coastal waters compared to inland waters experiencing higher inputs of terrestrial organic matter.^8,49^

Overall, ARINT models using optical proxies of *P*_π_ led to large (21–65%) improvements in DOC prediction errors for five data sets (*SUEZ, Horsens, Everglades, Yukon Lakes, S. America*) but little improvement (1–4%) for *Australia*, *Congo*, and *Alaska Rivers* (Figures S5–S12). However, the relative lack of improvement for the latter data sets is expected due to the strong prior correlations between DOC and *A*_254_ (*R* ^2^ = 0.94, 0.98, and 1.0 for *Australia*, *Congo*, and *Alaska Rivers*, respectively) (Table S4).

The Pan-Arctic model^49^ predicts DOC from *S*_275_295_ and *a*_350_ using tunable constants *C* and *M*. For the current data sets, *C* ranged between −1.0 and +1.3 while *M* ranged between 0.04 and 0.07 (Tables S4–S5 and Figures S5–S12). For *Yukon Lakes*, the Pan-Arctic model produced the most accurate DOC predictions among all tested models, with a 58% reduction in error. However, for all other data sets, the Pan-Arctic model produced larger prediction errors than ARINT models, and in five cases, it performed worse than the base model (Table S4). Note that the Pan-Arctic model was not tested with the *SUEZ* data set due to the missing absorbance spectra.

The global DOC_UV_ model improved DOC predictions relative to the base model for *Yukon Lakes* (*R* ^2^ = 0.66, RMSE = 7.1 mg L^–1^), but produced poorer predictions than the base model for all other data sets. In the case of *Congo*, the DOC_UV_ model produced very high error residuals and negative *R* ^2^, which occurs when a regression model represents a worse fit to the data than a horizontal line (Figure S7). Among local DOC_LS_ models, coefficient Φ ranged from 604–3156 compared to 1507 in the global model, while *DOCcor* ranged from −317 to 300 compared to 32.2 in the global model. As expected, the locally calibrated DOC_LS_ models made more accurate DOC predictions than the global model, but improvements relative to the base model were still only observed for *Yukon Lakes* and *Everglades* (Tables S4–S5 and Figures S5–S12).

Since the Pan-Arctic, DOC_UV_ and DOC_LS_ models were each developed from data sets consisting of predominantly marine samples,^44,49^ the higher error residuals produced by these models illustrate the inherent risks of applying empirical models developed from oceanic data sets to predict DOC concentrations in inland waters. The ARINT model results further suggest that in inland waters, the ability to sensitively detect variations in DOC aromaticity is key to accurately estimating DOC concentrations.

### 3.5 Improving DOC Predictions from Absorbance Measurements

In aquatic systems dominated by terrestrial DOM, there are typically tight correlations between CDOM absorption and DOC, allowing DOC concentrations to be accurately predicted from *A*_254_.^73^ However, numerous studies show that these parameters diverge in concert with decreasing hydrologic connectivity to the landscape. In impounded waterbodies, sustained photoirradiation reduces the overall diversity of DOM molecular formulas by diminishing the abundances of highly aromatic compounds while producing a smaller number of lower-molecular-weight compounds, including many unsaturated molecules but also some phenolic compounds.^7,74,75^ Phytoplankton primary productivity produces DOC molecules that can be consumed or modified during secondary microbial production, with a high overlap in molecular formulas between photolabile versus biolabile molecules.^76^ High diversities in aromatic structures result in the decoupling of absorbance and DOC, preventing the accurate prediction of DOC concentration from *A*_254_ alone.^30,32,73,75^ This phenomenon is extensively described and presents a significant hindrance to predicting DOC concentrations from optical measurements in ecological, biogeochemical, and remote-sensing studies.

Equation 6 extends the prediction of DOC concentrations from *A*_254_ by accounting for interactions between light absorption and aromaticity. The first term (*b*_1_ × *A*_254_) represents the prevailing relationship between DOC and absorbance across a data set, whereby absorbance increases in direct proportion to the number of carbon atoms. The second term (*b*_2_ × *A*_254_ × *P*_π_) is negative. This term reduces predicted DOC relative to the prevailing relationship, with the smallest reductions in samples with low aromaticity and the largest in samples with high aromaticity. When *P*_π_ = ARIX, this term probably compensates for the situation that the conjugated polyphenolic structures represented by the ARIX numerator emit significantly more light per carbon atom than the simpler phenolic structures represented by the ARIX denominator.

Equation 7 allows estimation of the variability in molar absorptivities of DOC molecules using ε ∝ 1/(*b*_1_ + *b*_2_ *P*_π_). When *b*_1_ is large, there is a relatively slow increase in A_254_ with increasing DOC, indicating absorption by lower-molecular-weight CDOM. Thus, *Yukon Lakes* and *Everglades* with *b*_1_ ∼ 1.0 are expected to be dominated by lower molecular weight chromophores compared to *Alaska Rivers* and *Horsens* with *b*_1_ ∼ 0.2–0.4. The range of *b*_2_ *P*_π_ relative to *b*_1_ indicates the influence of aromaticity on the proportionality between *A*_254_ and DOC. For all proxies in the studied data sets, the full range of values taken by *b*_2_ *P*_π_/*b*_1_ was approximately −0.1 to −0.8 (Table S6). When *b*_2_ *P*_π_ was small relative to *b_1_* across the entire data set (e.g., *Alaska Rivers*), or when *b*_2_ *P*_π_/*b*_1_ spanned a small range (e.g., *Australia*), then ε was effectively constant, and little improvement was obtained relative to predicting DOC from *A*_254_ alone. Conversely, *b*_2_ *P*_π_/*b*_1_ was relatively large and variable in the data sets that benefited the most from ARINT models (*Everglades, Yukon Lakes*, Table S6).

Further research is needed to test the ARINT algorithms more widely and examine how different factors affect the prediction of SUVA and DOC concentrations from DOM optical properties. It is especially important to investigate how predictions are impacted by seasonal and temporal variation, to quantify potential interferences and matrix effects, and to isolate natural sources of variability from instrumental sources. Also, since optical measurements do not detect colorless DOC, predictions from ARINT models about the size of this fraction are especially uncertain, and experimental validations are warranted. While prior research indicates that the linear correlation between SUVA and PARIX is usually preserved during physical–chemical treatment,^19^ further studies are warranted to identify potential limitations and interferences in a water treatment context.

### 3.6 Benefits for Water Quality Monitoring

Global surface waters face a changing climate with greater variability in both the quantity and quality of DOM. Warmer temperatures are increasing rates of litter decomposition in soils and rates of biological production in water at the same time as changes in land use and altered rainfall patterns are changing hydrological regimes and the connectivity between landscapes and DOM.^77,78^ Decreasing hydrological connectivity reduces correlations between DOC and spectroscopic measurements and decreases the accuracy of predicting DOC concentrations from in situ measurements or from remotely sensed imagery.^79^ Especially in inland systems, the accurate retrieval of absorption coefficients from satellite data is often challenged by complex atmospheric and optical conditions combined with seasonal variation and episodic events that cause rapid changes in DOC characteristics over relatively small temporal and spatial scales.^8,80^ Due to the link between aromaticity and chemical reactivity, any lack of predictability in surface water composition negatively affects drinking water treatment by increasing the risk of chemical over- or underdosing.^19^

The relationships revealed in this study can be used to improve the prediction of DOC aromaticity and concentration from spectroscopic measurements obtained in inland waterbodies exhibiting decoupling between DOC concentrations and absorption coefficients. New *in situ* spectroscopic instruments could leverage these results to deliver currently missing data and provide real-time predictions of DOC concentration, reactivity, and fate. Such instruments could simplify ground-truthing of remote-sensing algorithms in optically complex inland waters. Further applications include drinking water treatment, whereby real-time optical data could be used to adjust chemical doses in response to changing DOC composition, facilitating the sustainable removal of DOC compounds and their associated micropollutants.

## Supplementary Material



## Data Availability

The data needed to reproduce these results are available in the Dryad database (10.5061/dryad.x69p8czt8). MATLAB code for extracting ARIX and other fluorescence indices from EEMs is available via the pickpeaks function of the drEEM software package at https://dreem.openfluor.org/.

## Notes

Additional experimental details and methods, SUVA and DOC predictions for individual data sets (plots and fitting statistics), model inference criteria, and assumptions and results of the sensitivity analysis for predicting SUVA from ARIX (PDF)
