# Supplementary material for: Prediction of Dissolved Organic Carbon Concentrations in Inland Waters Using Optical Proxies of Aromaticity
Source: Environ Sci Technol. 2025 Jul 30;59(31):16430–42. doi: 10.1021/acs.est.5c05408 (PMC12355942; doi:10.1021/acs.est.5c05408)
Supplement: Supplementary file 1 [file es5c05408_si_001.pdf]

Prediction of dissolved organic carbon concentrations in inland waters using optical proxies of aromaticity.

Supplementary Information

Kathleen R. Murphy[1,2]*†

[1]Department of Architecture and Civil Engineering, Chalmers University of Technology; Gothenburg, 41298 Sweden.

[2]Department of Building and Environmental Technology, Lund University; Lund, 22363 Sweden.

*Corresponding author. Email: murphyk@chalmers.se

This document consists of 21 pages containing 14 figures and 6 tables.

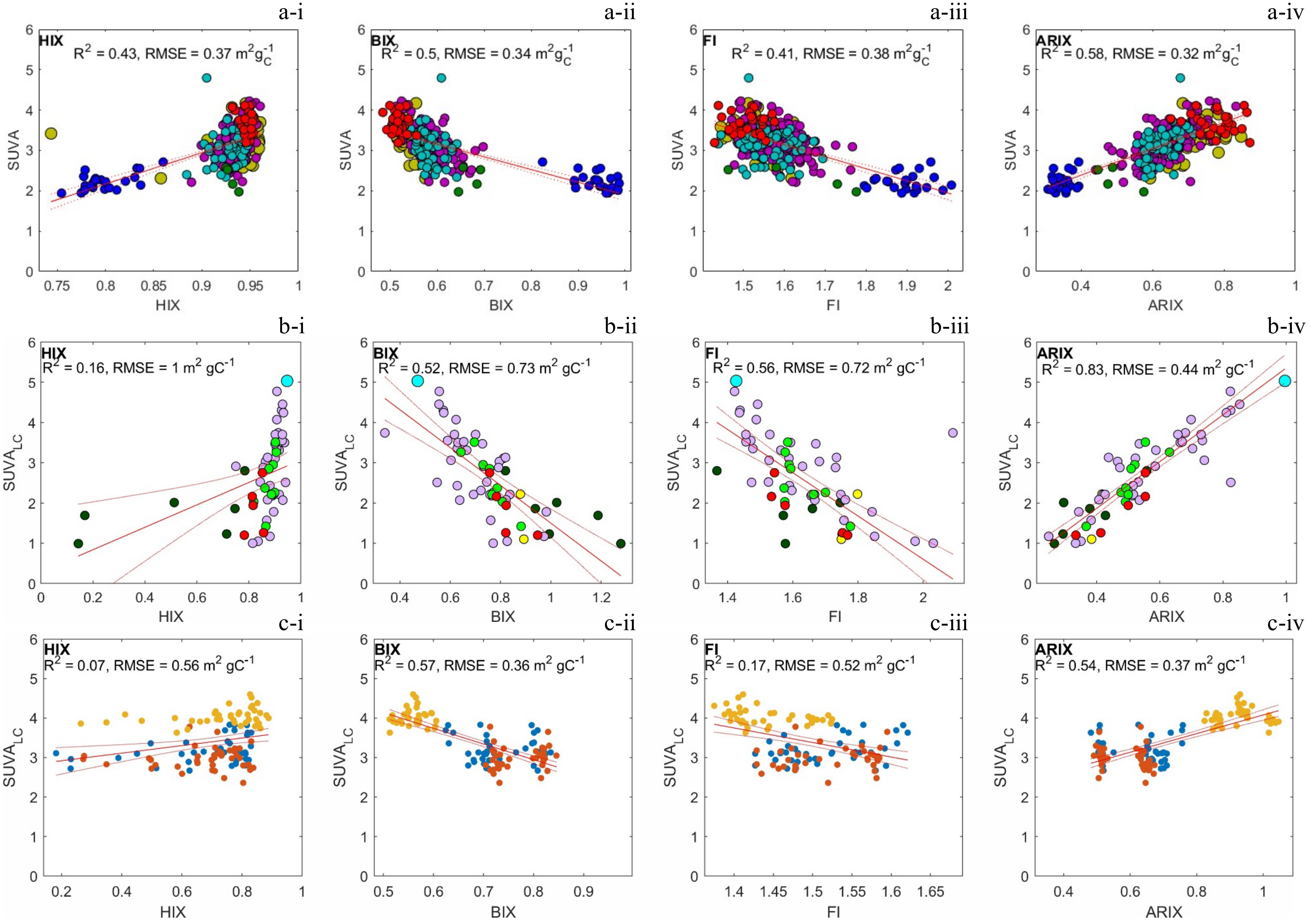

Fig. S1: Robust linear regressions of SUVA ($m^2 g_C^{-1}$) on fluorescence indices: i) HIX, ii) BIX, iii) FI and iv) ARIX for (a) River samples from the *Horsens* dataset, and (b) drinking water sources at various stages of treatment in the *SUEZ* dataset; (c) *Australia* dataset. Colour codes correspond to sample locations as per the main manuscript.

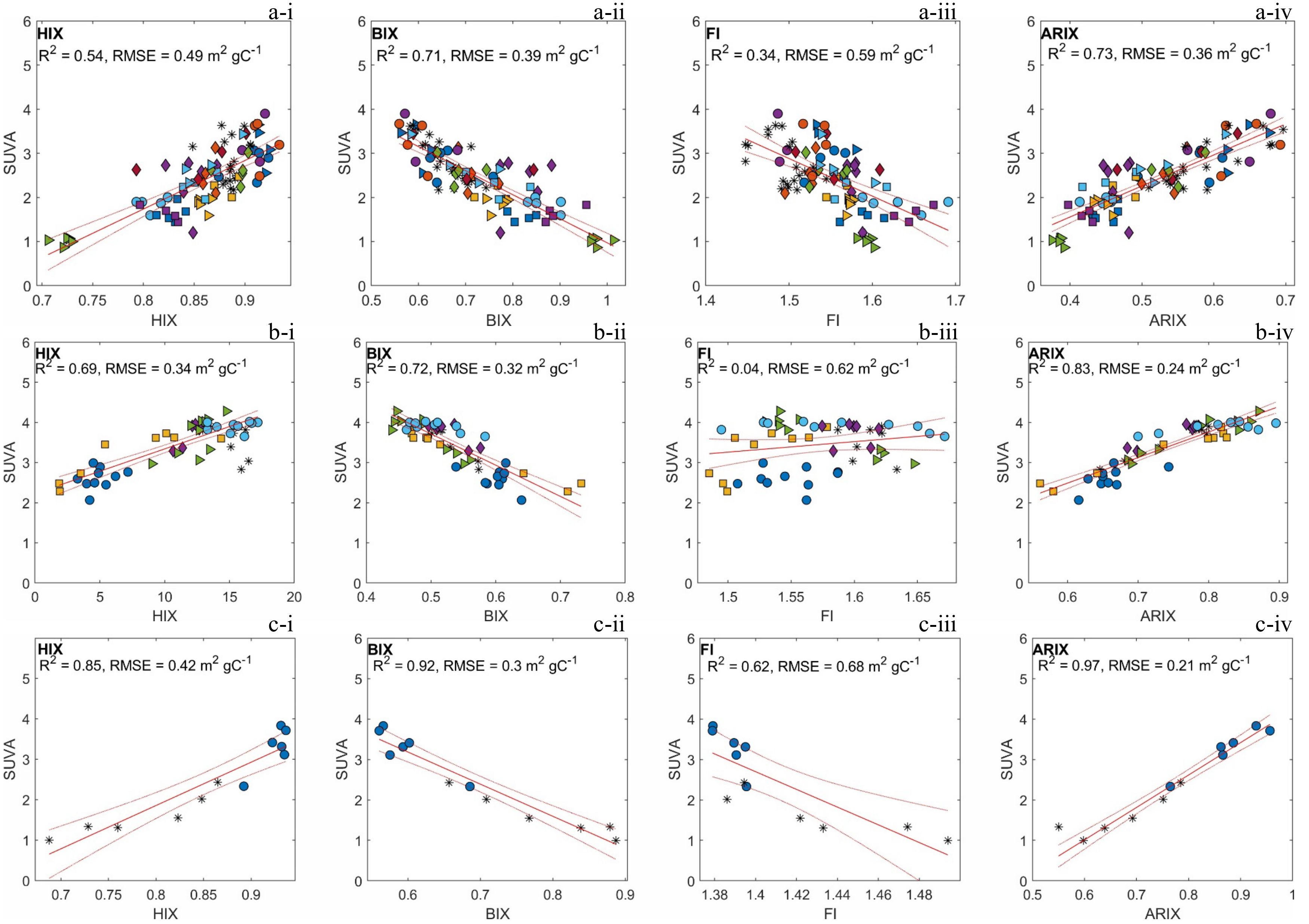

Fig. S2: Robust linear regressions of SUVA ($m^2 gC^{-1}$) on fluorescence indices: i) HIX, ii) BIX, iii) FI and iv) ARIX for (a) samples from the *Yukon Lakes* dataset; (b) samples from the *Alaska Rivers* dataset; and (c) samples from the *Everglades* dataset. Colour codes correspond to sample locations as per the main manuscript.

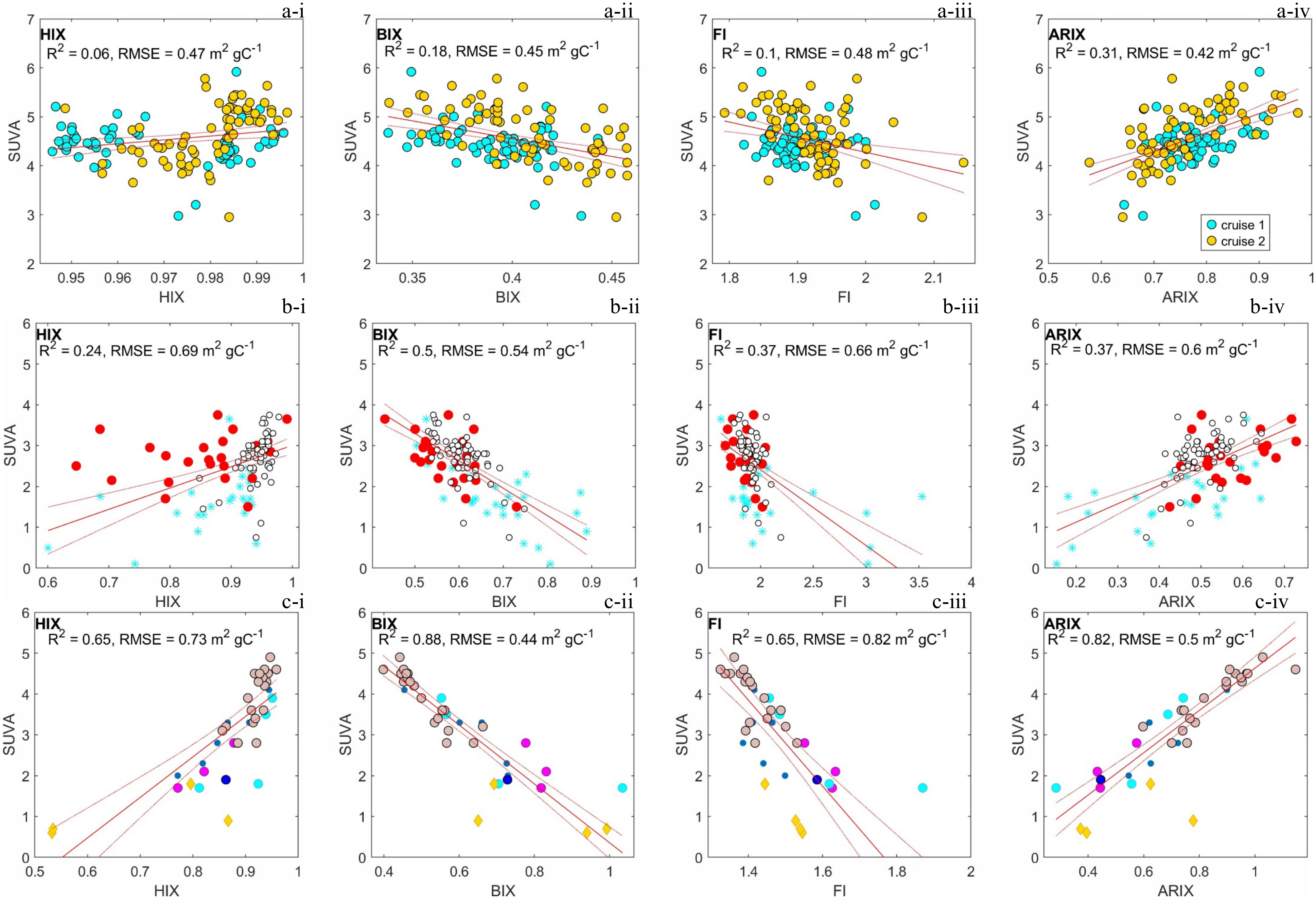

Fig. S3: Robust linear regressions of SUVA ($m^2 g_C^{-1}$) on fluorescence indices: i) HIX, ii) BIX, iii) FI and iv) ARIX for samples from (a) the *Congo* dataset, (b) the *S. America* dataset, (c) the *Isolates* dataset.

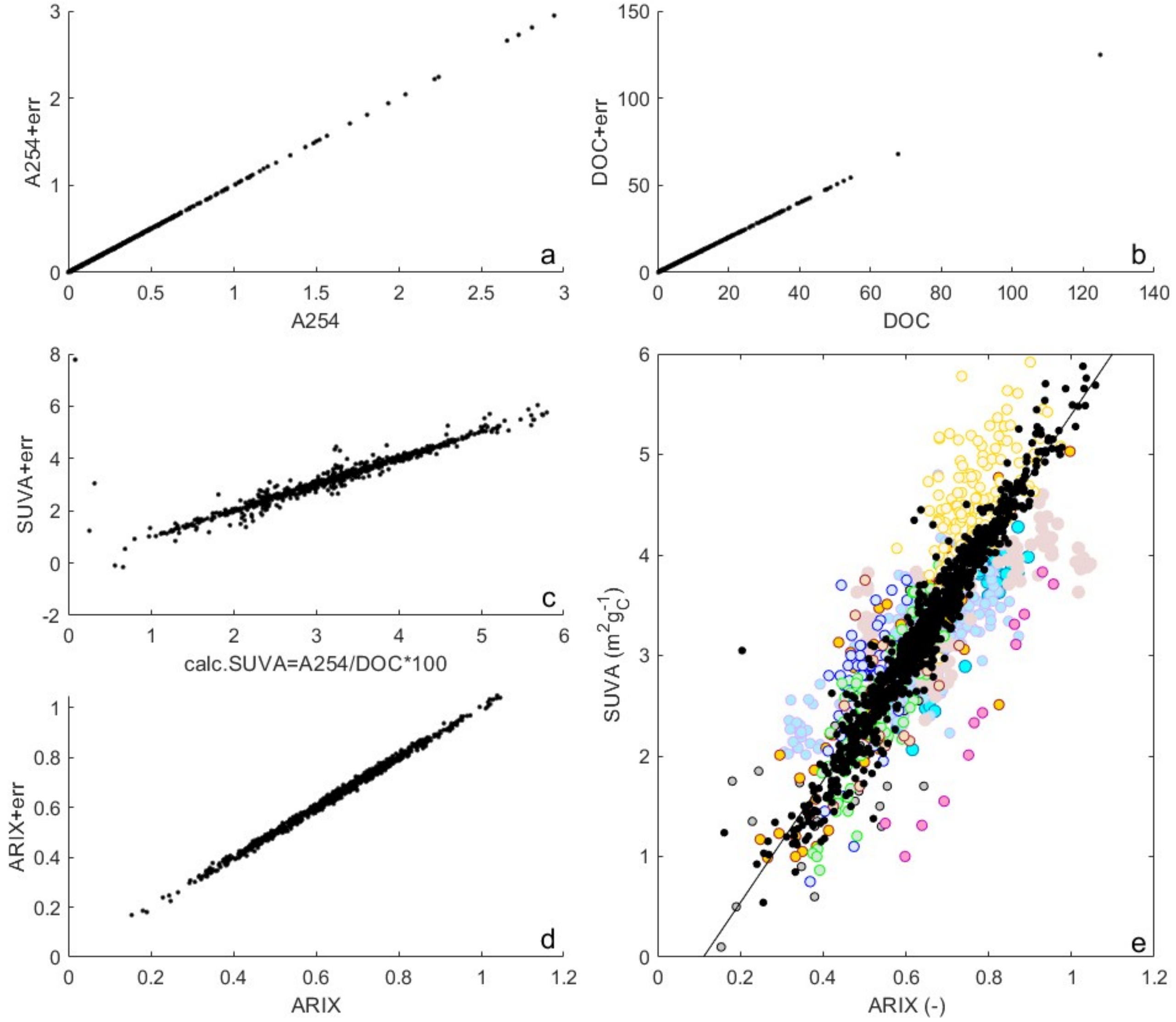

Fig. S4: Error sensitivity of ARIX vs SUVA relationship. Refer to Table S3 for model assumptions; note that the *Everglades* dataset (n=12) was excluded. (a) measured A254 vs simulated A254 (cm^{-1}); (b) measured DOC vs simulated DOC (mgL^{-1}); (c) error free SUVA vs SUVA with error, i.e. (A254+err)/(DOC+err) x 100 ($m^2g_C{}^{-1}$); (d) measured ARIX vs simulated ARIX (e) Measured data (pale circles) with overlaid simulated data representing SUVA with error vs ARIX with error in a single run of the model (black dots). RMSE for the observed data relative to Eq. 3, which is depicted by the straight line in panel (e), is 0.598 while the median RMSE for 100 simulations was 0.194 $m^2g_C{}^{-1}$, indicating that random errors in the model account for ~32.4% of total observed error in the measured datasets.

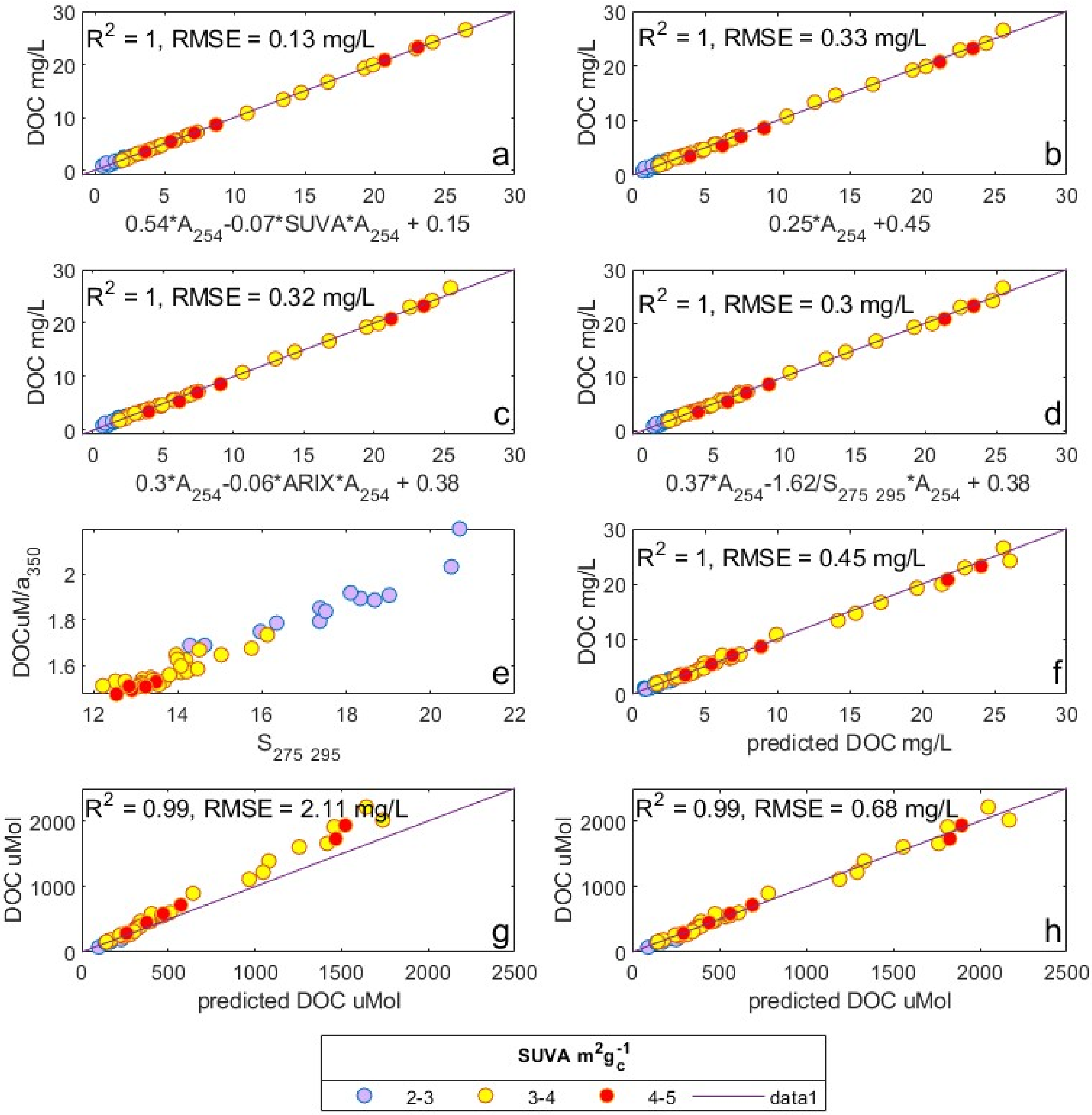

Fig. S5: Prediction of DOC concentrations from optical measurements for *Alaska Rivers*. a: ARINT multilinear model with A_{254} interacting with SUVA; b: simple linear regression on A_{254}; c: ARINT model with A_{254} interacting with ARIX; d: ARINT model with A_{254} interacting with S_{275_295}; e: Pan-Arctic model based on a_{350} and S_{275_295} after Gonçalves-Araujo et al. 2023; f: measured vs predicted DOC using the model in e. g: Global DOC_{UV} model based on a_{275}, S_{275_295} and $S_{380\text{-}443}$ after Yan et al. 2025; h: optimised DOC_{LS} model after Yan et al. 2025. Relative to the base model in panel b, only the models in panels a, c and d produce significantly improved DOC predictions according to Akaike's and Bayesian information criteria.

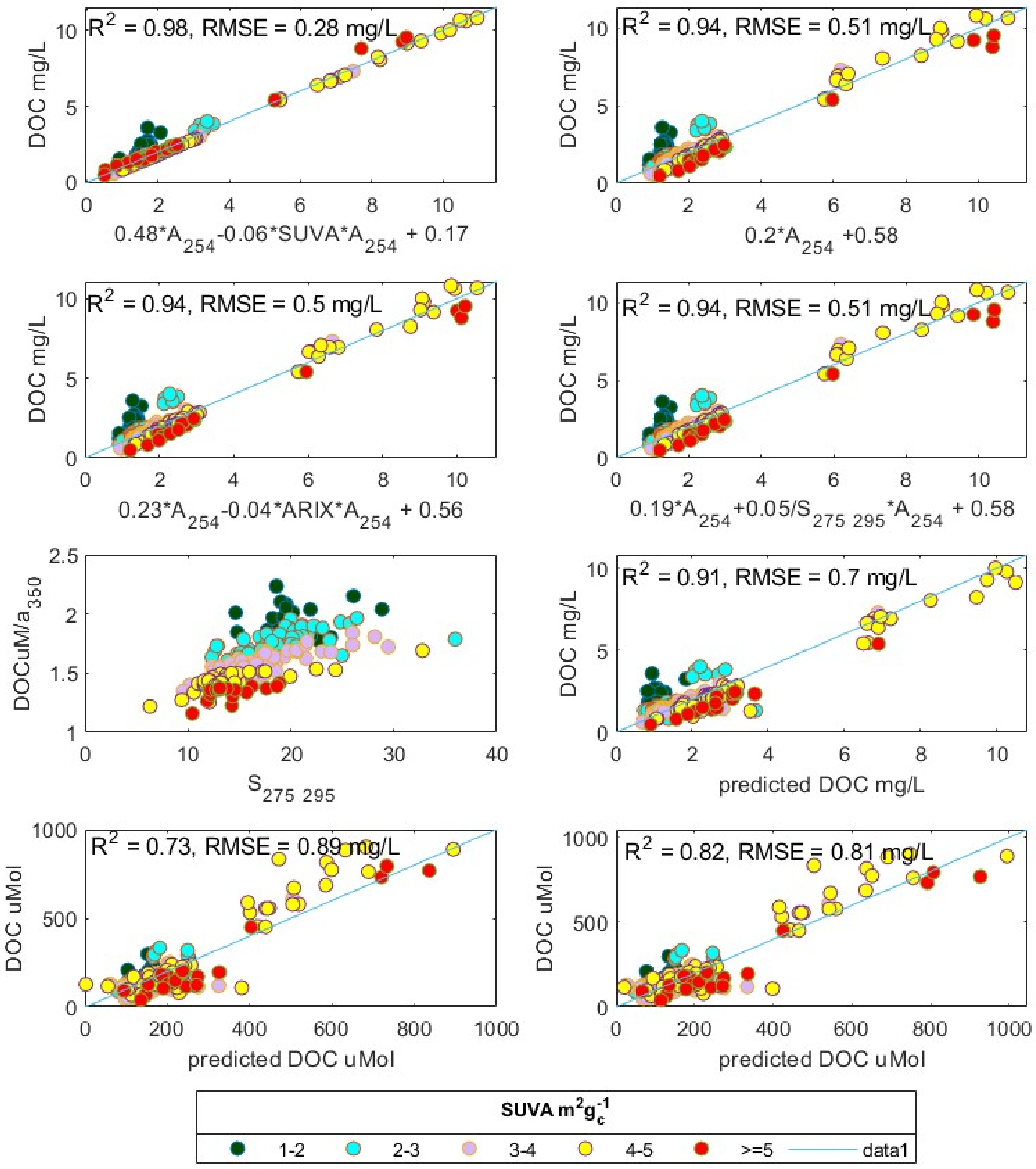

Fig. S6: Prediction of DOC concentrations from optical measurements for *Australia*. a: ARINT multilinear model with A_{254} interacting with SUVA; b: simple linear regression on A_{254}; c: ARINT model with A_{254} interacting with ARIX; d: ARINT model with A_{254} interacting with S_{275_295}; e: Pan-Arctic model based on a_{350} and S_{275_295} after Gonçalves-Araujo et al. 2023; f: measured vs predicted DOC using the model in e. g: Global DOC_{UV} model based on a_{275}, S_{275_295} and $S_{380-443}$ after Yan et al. 2025; h: optimised DOC_{LS} model after Yan et al. 2025. Relative to the base model in panel b, only the models in panel a and c produce significantly improved DOC predictions according to Akaike's and Bayesian information criteria.

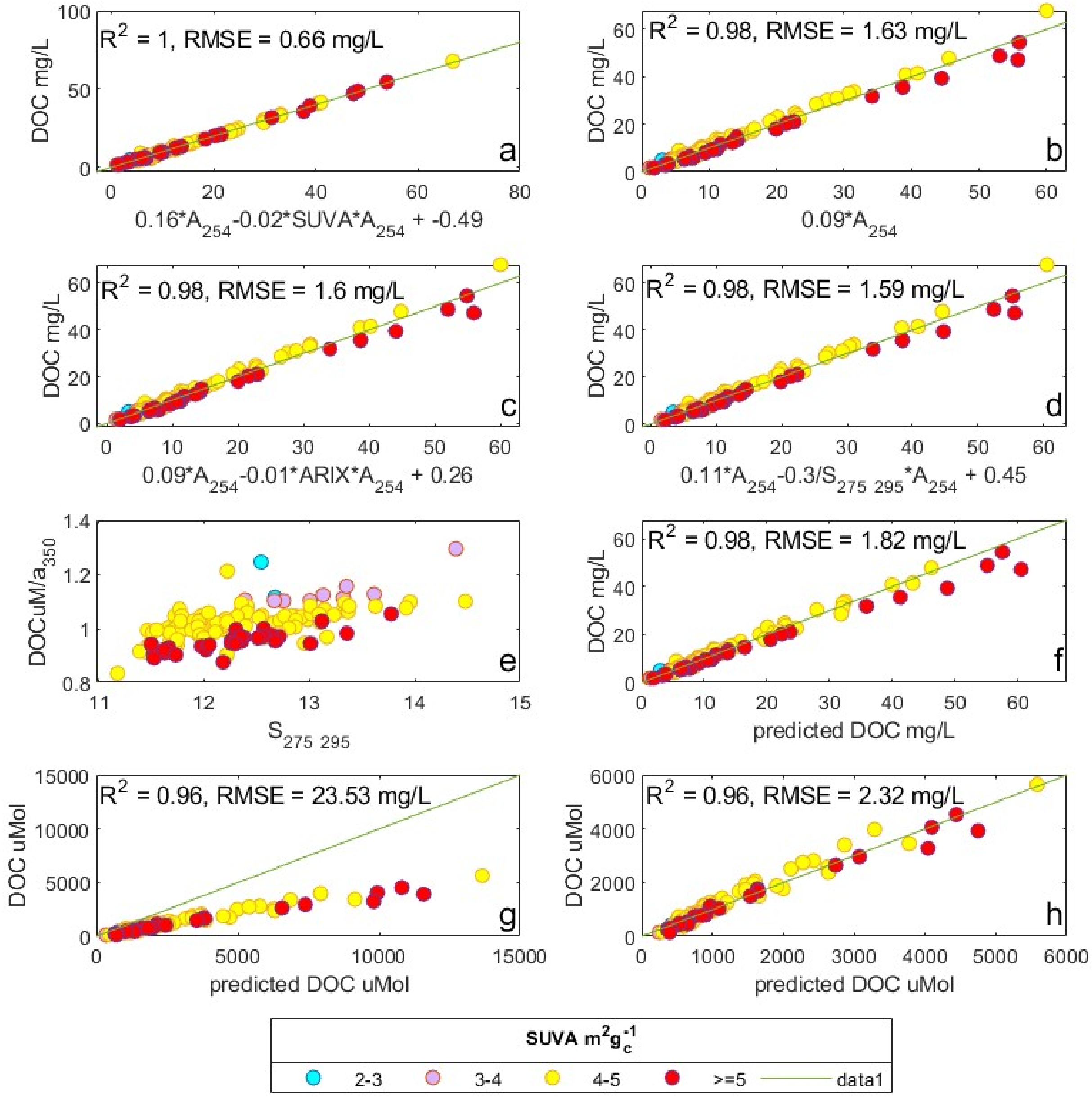

Fig. S7: Prediction of DOC concentrations from optical measurements for *Congo*. a: ARINT multilinear model with A_{254} interacting with SUVA; b: simple linear regression on A_{254}; c: ARINT model with A_{254} interacting with ARIX; d: ARINT model with A_{254} interacting with S_{275_295}; e: Pan-Arctic model based on a_{350} and S_{275_295} after Gonçalves-Araujo et al. 2023; f: measured vs predicted DOC using the model in e. g: Global DOC_{UV} model based on a_{275}, S_{275_295} and $S_{380\text{-}443}$ after Yan et al. 2025; h: optimised DOC_{LS} model after Yan et al. 2025. Relative to the base model in panel b, only the models in panel a, c and d produce significantly improved DOC predictions according to Akaike's and Bayesian information criteria.

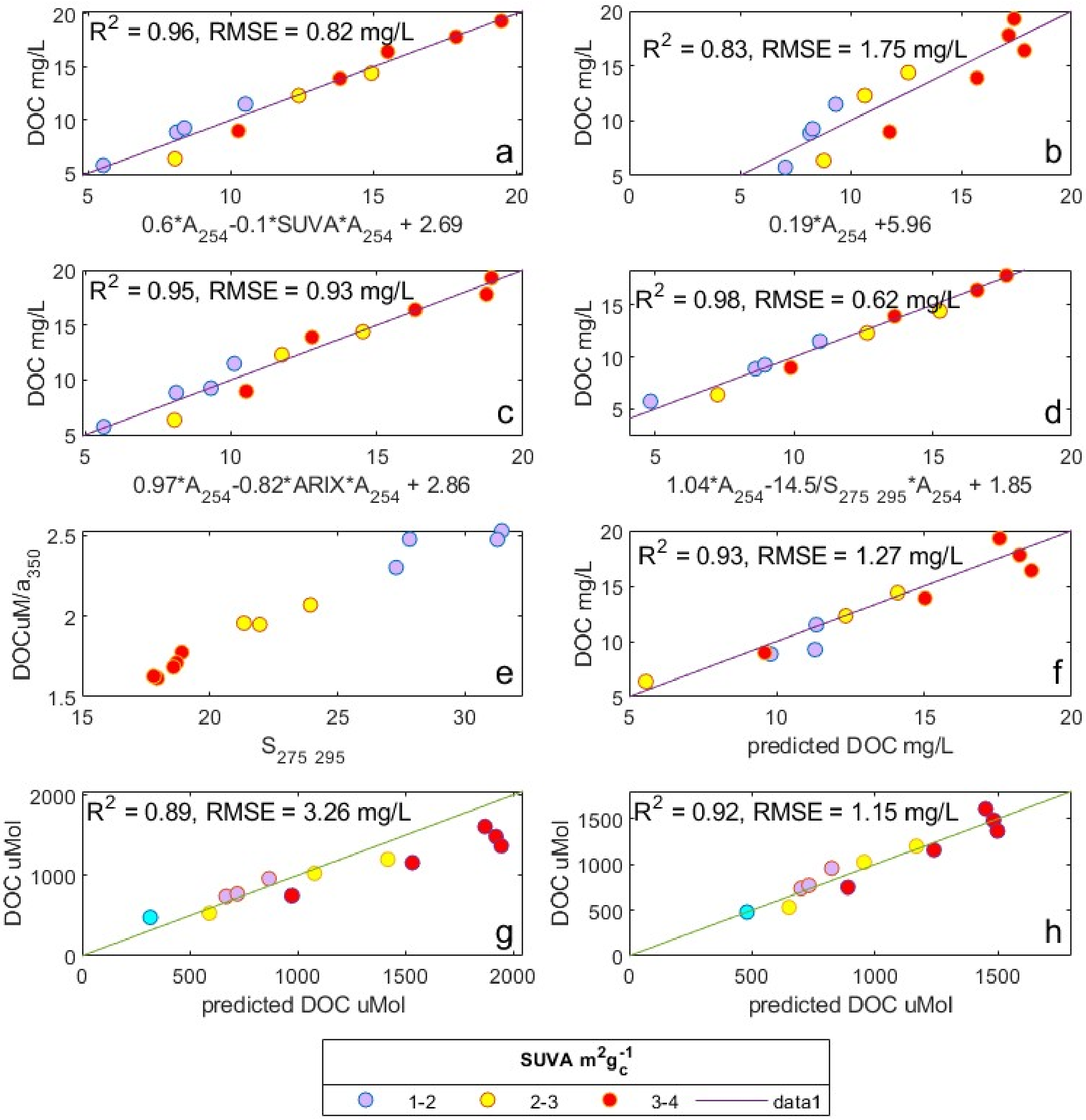

Fig. S8: Prediction of DOC concentrations from optical measurements for *Everglades*. a: ARINT multilinear model with A_{254} interacting with SUVA; b: simple linear regression on A_{254}; c: ARINT model with A_{254} interacting with ARIX; d: ARINT model with A_{254} interacting with S_{275_295}; e: Pan-Arctic model based on a_{350} and S_{275_295} after Gonçalves-Araujo et al. 2023; f: measured vs predicted DOC using the model in e. g: Global DOC_{UV} model based on a_{275}, S_{275_295} and $S_{380\text{-}443}$ after Yan et al. 2025; h: optimised DOC_{LS} model after Yan et al. 2025. Except for panel g, all models provide significantly better DOC predictions than the base model in panel b, according to Akaike's and Bayesian information criteria.

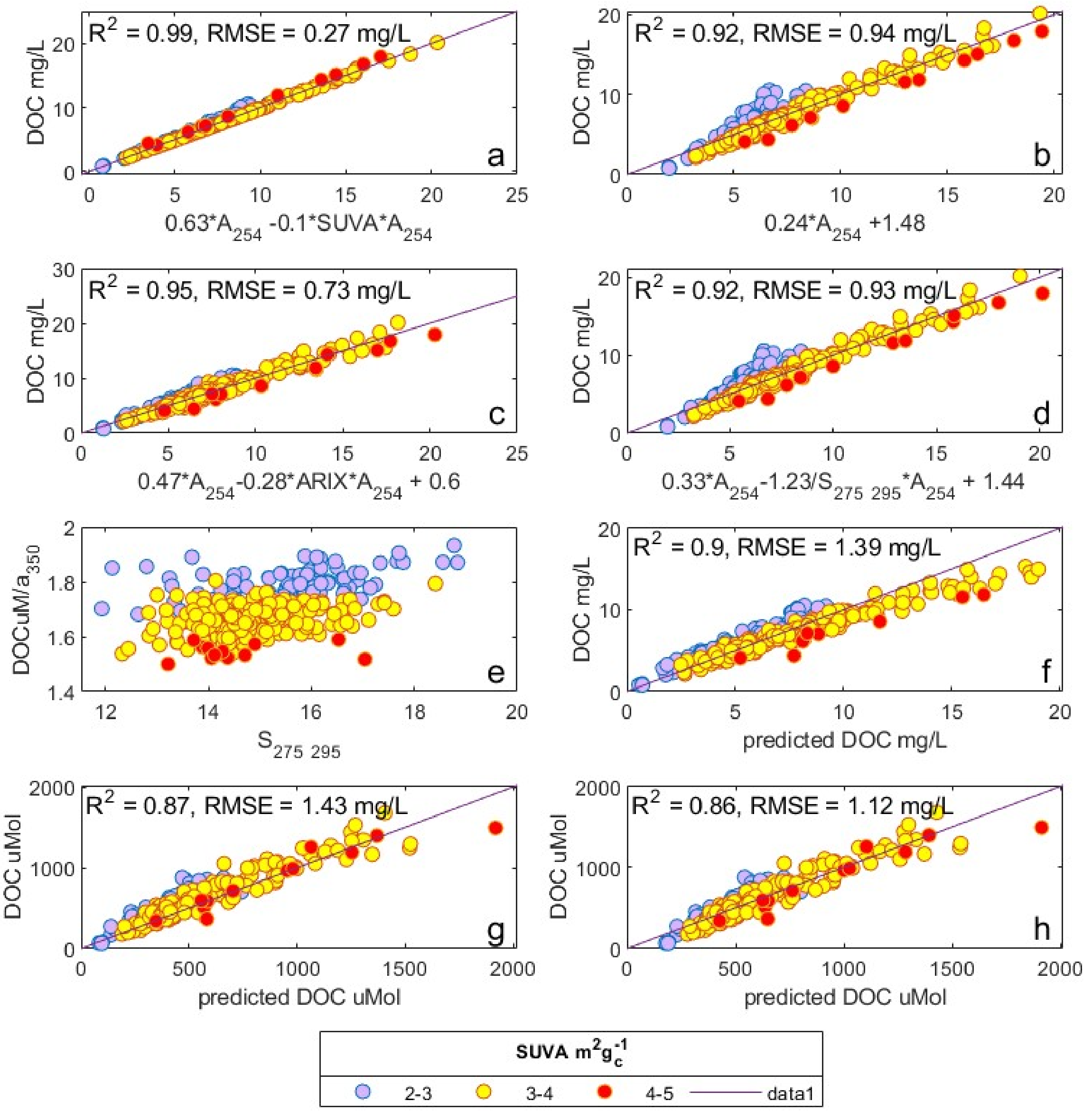

Fig. S9: Prediction of DOC concentrations from optical measurements for *Horsens*. a: ARINT multilinear model with A_{254} interacting with SUVA; b: simple linear regression on A_{254}; c: ARINT model with A_{254} interacting with ARIX; d: ARINT model with A_{254} interacting with S_{275_295}; e: Pan-Arctic model based on a_{350} and S_{275_295} after Gonçalves-Araujo et al. 2023; f: measured vs predicted DOC using the model in e. g: Global DOC_{UV} model based on a_{275}, S_{275_295} and $S_{380\text{-}443}$ after Yan et al. 2025; h: optimised DOC_{LS} model after Yan et al. 2025. All models provide significantly better DOC predictions than the base model in subplot b, according to Akaike's and Bayesian information criteria. Relative to the model in panel b, only the models in panel a, c and d produce significantly improved DOC predictions according to Akaike's and Bayesian information criteria.

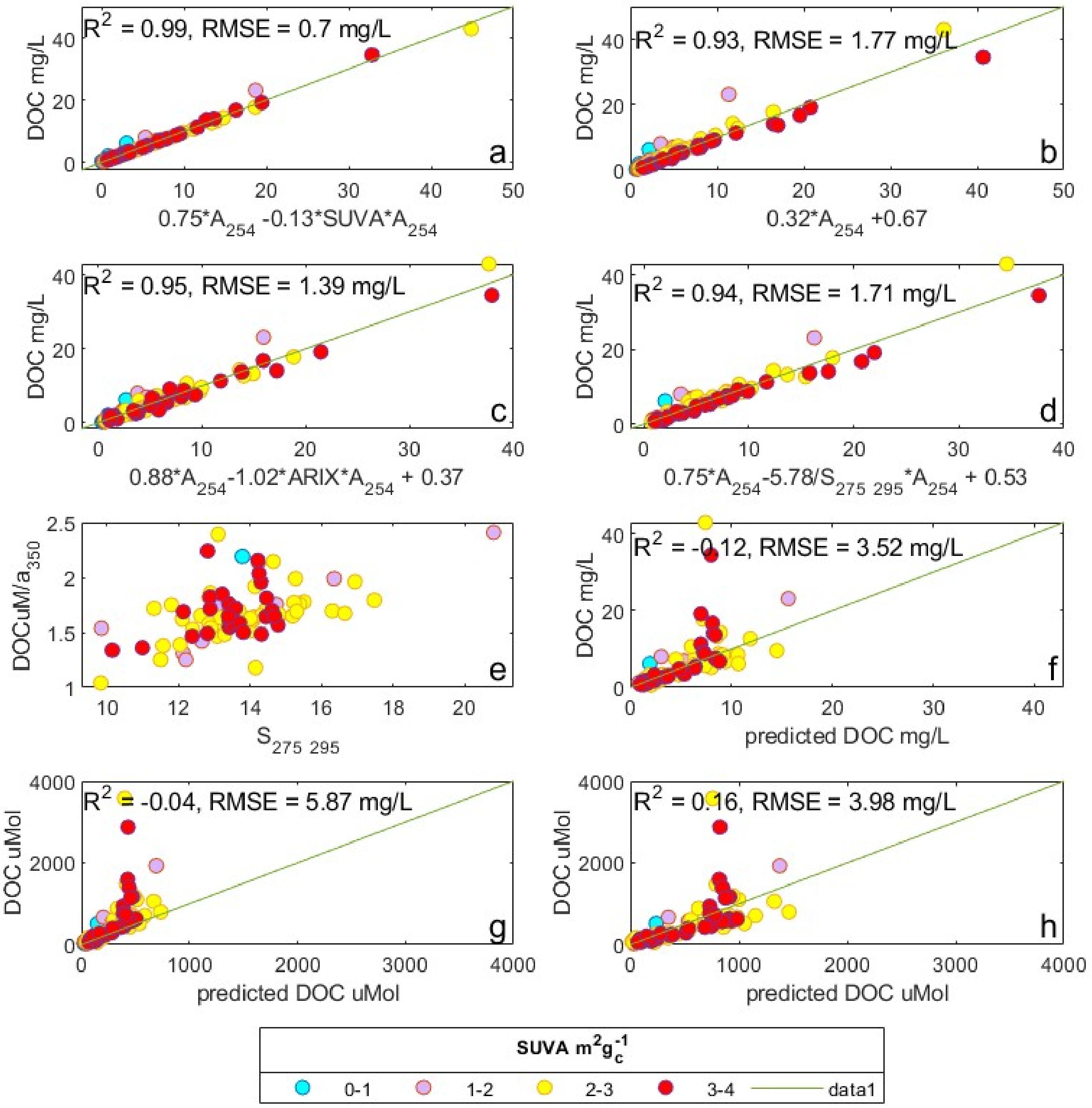

Fig. S10: Prediction of DOC concentrations from optical measurements for *S. America*. a: ARINT multilinear model with A_{254} interacting with SUVA; b: simple linear regression on A_{254}; c: ARINT model with A_{254} interacting with ARIX; d: ARINT model with A_{254} interacting with S_{275_295}; e: Pan-Arctic model based on a_{350} and S_{275_295} after Gonçalves-Araujo et al. 2023; f: measured vs predicted DOC using the model in e. g: Global DOC_{UV} model based on a_{275}, S_{275_295} and $S_{380-443}$ after Yan et al. 2025; h: optimised DOC_{LS} model after Yan et al. 2025. Relative to the model in panel b, only the models in panels a, c and d produce significantly improved DOC predictions according to Akaike's and Bayesian information criteria.

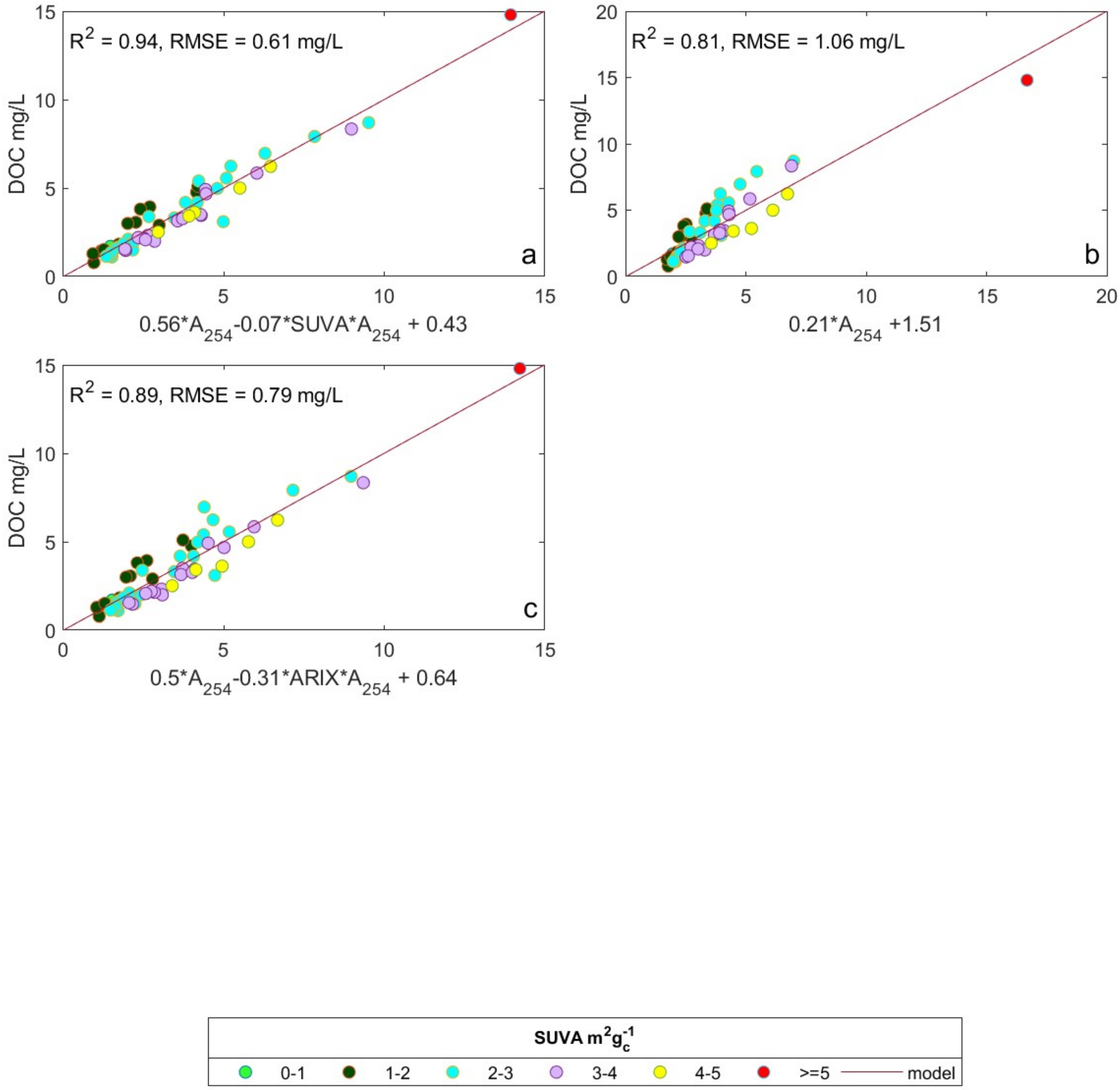

Fig. S11: Prediction of DOC concentrations from optical measurements for *SUEZ*. a: ARINT multilinear model with A_{254} interacting with SUVA; b: simple linear regression on A_{254}; c: ARINT model with A_{254} interacting with ARIX. The models in panels a and c each provide significantly better DOC predictions than the base model in panel b, according to Akaike's and Bayesian information criteria. Models requiring absorbance spectral slopes are unavailable due to missing absorbance spectra.

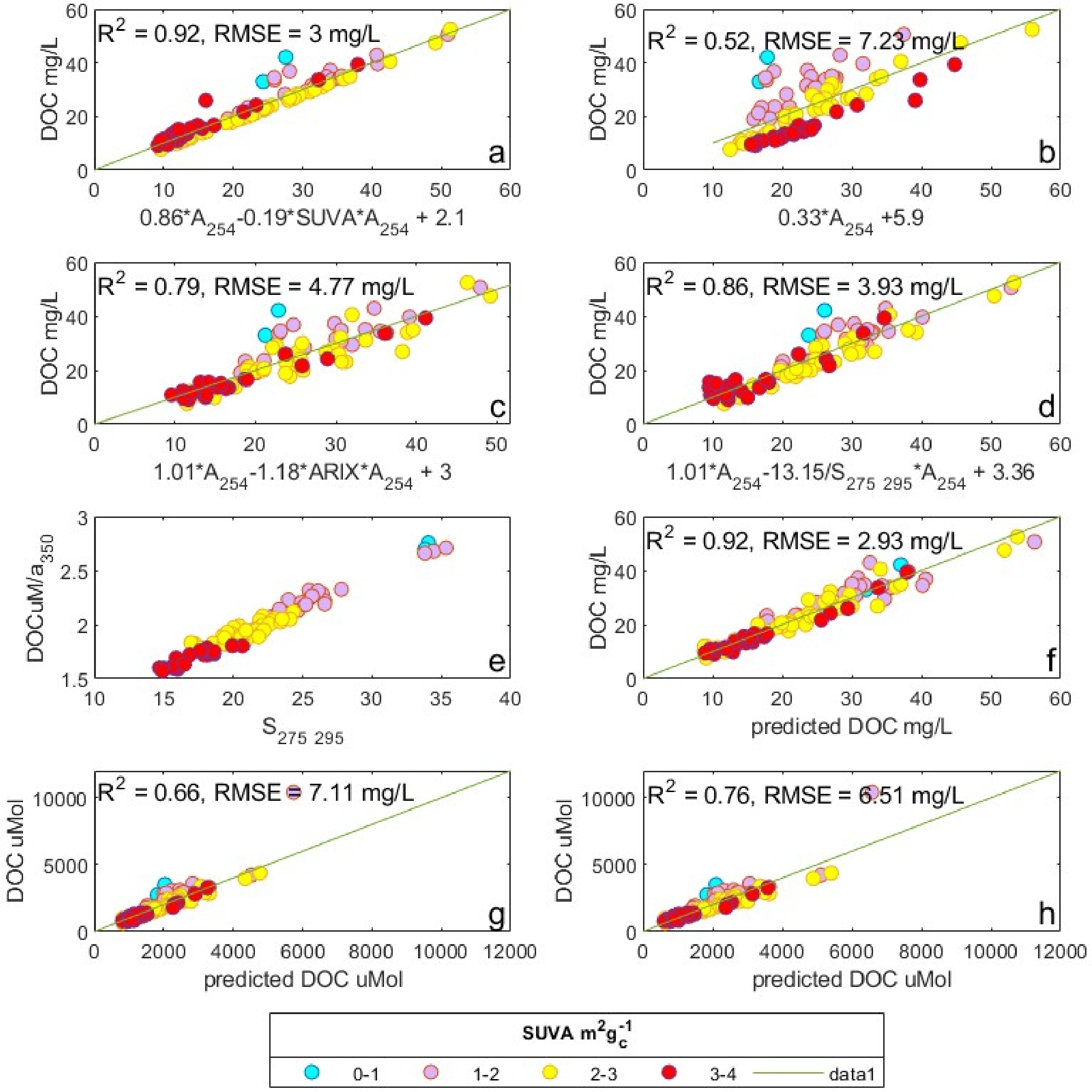

Fig. S12: Prediction of DOC concentrations from optical measurements for *Yukon Lakes.* a: ARINT multilinear model with A_{254} interacting with SUVA; b: simple linear regression on A_{254}; c: ARINT model with A_{254} interacting with ARIX; d: ARINT model with A_{254} interacting with S_{275_295}; e: Pan-Arctic model based on a_{350} and S_{275_295} after Gonçalves-Araujo et al. 2023; f: measured vs predicted DOC using the model in e. g: Global DOC_{UV} model based on a_{275}, S_{275_295} and $S_{380\text{-}443}$ after Yan et al. 2025; h: optimised DOC_{LS} model after Yan et al. 2025. All models provide significantly better DOC predictions than the base model in panel b, according to Akaike's and Bayesian information criteria.

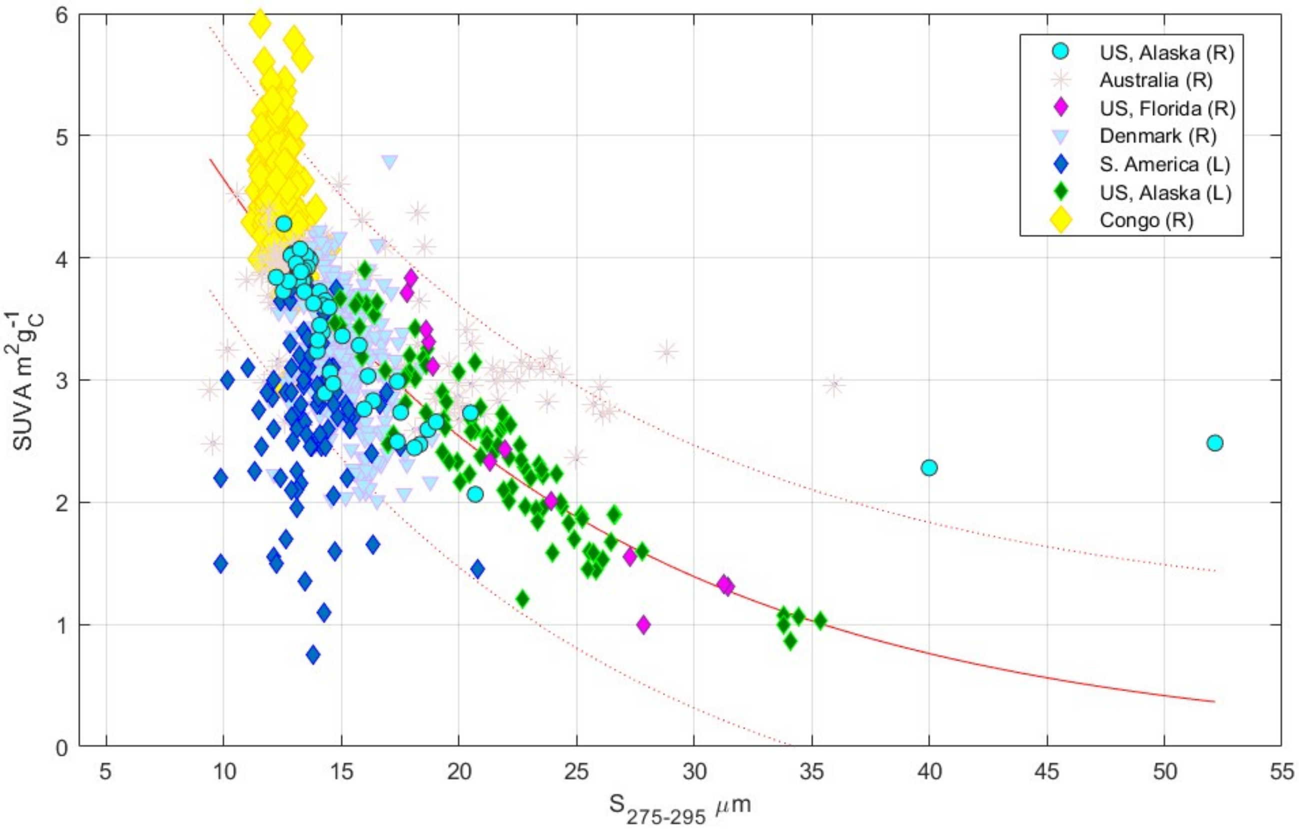

Fig. S13: Regression of SUVA upon spectral slope S_{275_295} for the wholewater datasets. The non-linear regression curve was obtained using bisquare robust fitting and is derived from five of the seven datasets named in the legend (*S. America* and *Australia* were excluded). It has the equation SUVA=8.48 x exp(-0.060*S_{275_295}) and the dotted lines show the 95% prediction bounds of the fitted curve (RMSE = 0.54, R^2 = 0.57, N = 712).

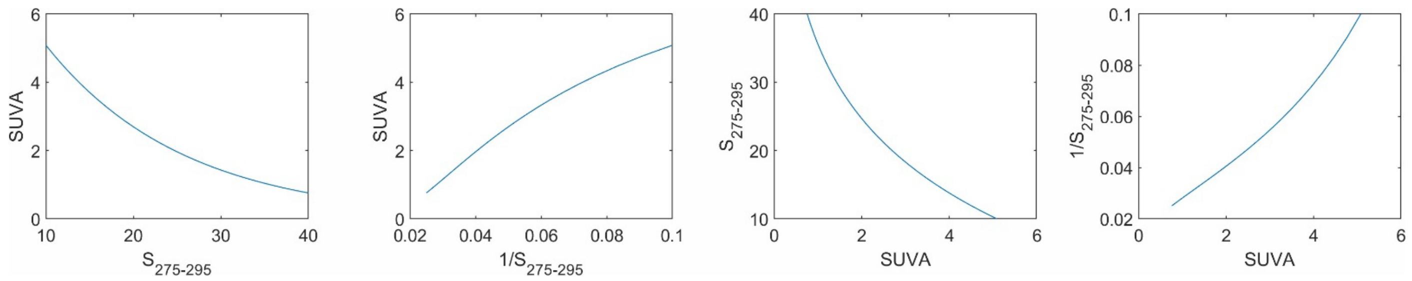

Fig. S14: Modelled correlations between SUVA ($m^2g_c^{-1}$) and spectral slope S_{275_295} (μm^{-1}) or inverse spectral slope, $1/S_{275\_295}$. In each case the model corresponds to the equation in Fig S13.

Table S1: Metadata and summary results for analysed datasets. Fluorescence measurements were on whole-water samples (W) or DOM isolates (Is). Methods are coded for SUVA measurement (S-epa: A_{254}/DOC (USEPA method), S-lc: LC-OCD) and Fluorometer model (F-a: Horiba AquaLog, F-f2: Horiba Fluoromax-2, F-v: Varian Cary Eclipse, F-pe: Perkin-Elmer LS-50B). The regression equation predicts SUVA from ARIX.

Name	Bioregions	Sampling date	Sites	Methods and Variables	N	**Slope,** $\hat{m}$ ± SE	Data source
Alaska Rivers	Boreal North America	Summer, 5/2016-9/2016, 5/2017	6 rivers and streams	(W) S-epa, F-a	53	6.36 ± 0.41	(*1*)
Yukon Lakes	Boreal North America	Summer 2015, 2016; fall 2016, 2017; winter 2017	15 lakes	(W) S-epa, F-a	90	7.03 ± 0.45	(*2*)
Everglades	Subtropical North American wetland	4/2013	2 brackish rivers	(W) S-epa, F-a	12	8.01 ± 0.48	(*3*)
SUEZ	Europe: Continental, Mediterranean, China, Cameroon	Various 2016-2018	29 water treatment plants	(W) S-lc, F-a	58	5.80 ± 0.36	(*4*)
Horsens River	Continental Europe (Denmark)	Monthly 2001/08-2002/09	A river and tributaries	(W) S-epa, F-v	310	3.26 ± 0.16	(*5*)
Australia	Southeastern Australia	10/2019-3/2020	A river and tributaries	(W) S-lc, F-a	120	2.08 ± 0.19	(*6*)
Congo	Congo River, Africa	10/2013, 6/2014	A river and tributaries	(W) S-epa, F-a	131	3.71 ± 0.50	(*7*)
S. America	Brazil, Chile, Uruguay	Biweekly, 2/2010-5/2012	Headwater streams	(W) S-epa, F-pe	106	4.51 ± 0.58	(*8*)
Isolates	Extracts from USA, Antarctica, Pacific ocean.	1979-2015	diverse freshwater, groundwater, coastal	(Is) S-epa, F-a	37	4.50 ± 0.10	(*9*)

Table S2: Results of regressions (R^2 and RMSE) with SUVA or $SUVA_{LC}$ as the dependent variable and fluorescence indices. Models with best fit (lowest RMSE) for a particular dataset (row) are highlighted in bold text. All regressions used a robust algorithm to automatically exclude outliers except for ARIX, where § denotes results for an ordinary linear regression with no accommodation of outliers.

Dataset	Y-var	Statistic	ARIX	FI	β/α	BIX	HIX	HIX_{1999}	PARIX
Alaska rivers	SUVA	RMSE	**0.24**§	0.62	0.31	0.31	0.39	0.33	
Everglades	SUVA	RMSE	**0.21**	0.68	0.27	0.30	0.42	0.30	0.25
Horsens	SUVA	RMSE	**0.31**	0.38	0.34	0.34	0.37	0.37	
SUEZ	$SUVA_{LC}$	RMSE	**0.44**	0.72	0.74	0.73	1.00	1.00	0.64
S. America	$SUVA_{LC}$	RMSE	0.60§	0.66	**0.53**	**0.54**	0.69	0.67	
Yukon Lakes	SUVA	RMSE	**0.36**§	0.59	0.40	0.39	0.49	0.50	
Australia	$SUVA_{LC}$	RMSE	0.37	0.52	**0.36**	**0.36**	0.55	0.54	
Congo	SUVA	RMSE	**0.41**	0.48	0.45	0.45	0.47	0.46	
Isolates	SUVA	RMSE	0.50	0.82	**0.42**	0.44	0.73	0.71	**0.42**
Average RMSE (whole-waters)			**0.35**	0.58	0.43	0.43	0.55	0.52	
Average RMSE (all datasets)			**0.37**	0.61	0.42	0.43	0.57	0.54	
Alaska rivers	SUVA	R^2	**0.83**§	0.04	0.74	0.72	0.39	0.69	
Everglades	SUVA	R^2	**0.97**	0.62	0.93	0.92	0.85	0.92	0.96
Horsens	SUVA	R^2	**0.57**	0.41	0.50	0.50	0.43	0.45	
SUEZ	$SUVA_{LC}$	R^2	**0.83**	0.56	0.51	0.52	0.16	0.16	0.78
S. America	$SUVA_{LC}$	R^2	0.35§	0.35	**0.48**	**0.47**	0.16	0.33	
Yukon Lakes	SUVA	R^2	**0.73**§	0.34	0.70	0.71	0.54		
Australia	$SUVA_{LC}$	R^2	0.56	0.41	**0.64**	**0.70**	0.07	0.13	
Congo	SUVA	R^2	**0.30**	0.10	0.19	0.18	0.06	0.08	
Isolates	SUVA	R^2	0.82	0.65	**0.89**	0.89	0.65	0.66	**0.89**

Table S3: Model parameters in the error sensitivity analysis. For each parameter, error residuals were sampled randomly from a normal distribution with mean and standard deviation as reported below.

**Parameter**	**Detector**	**Random error model**	**Excluded sources of error/bias**
ARIX	Fluorescence	μ=0, SD = 0.01	Monochromator misalignment, emission spectra correction factors, inner filter correction factors (emission), stray light, cuvette variations.
SUVA	Absorbance, A_{254}	μ=0, SD = 0.003 cm^{-1}	Monochromator misalignment, stray light, cuvette variations, filtration errors.
SUVA	DOC	μ=0, SD = 0.1 mg/L	DOC calibration curve, cross-contamination between samples during measurement.

Table S4: Fits for models predicting DOC from optical measurements in whole-water datasets corresponding to Figures S5-S12. The prediction model is either the base model where DOC is predicted from A_{254} alone ($DOC = b_0 + b_1 \times A_{254}$), or else a multilinear Aromaticity Interaction (ARINT) Model, the empirical Pan-Arctic Model (*10*), or one of the empirical models of Yan et al. (*11*). In each ARINT Model, DOC is predicted from the multiple linear regression equation $DOC = b_0 + b_1 \times A_{254} + b_2 \times A_{254} \times P_\pi$ where P_π is an aromaticity proxy consisting of either ARIX, S_{275_295}, or SUVA. The Pan-Arctic Model is an empirical model of form $DOC = a_{350} \times$ 10^(b_0+($b_1 \times S_{275\_295}$) with b_0 = C and b_1 = M. The DOC_{UV} and DOC_{LS} models are of form $DOC = b_0\, a_{275}\, (S_{275\text{-}295} + 0.078\, S_{380\text{-}443} - 0.0084) + b_1$, with $b_0 = \Phi$ and $b_1 = DOC_{cor}$. Only positive values of δRMSE signify an improvement relative to the base model. Models producing statistically significant improvements are highlighted in bold; this determination is based on δRMSE together with information criteria in Table S5. Missing data are indicated by *n.d.*.

	SUVA quantiles			DOC=$b_0 + b_1$*A_{254}				Model coefficients						
Model	5%	50%	95%	b_0	b_1	R^2	RMSE (mg/L)	b_0	b_1	b_2	R^2	RMSE (mg/L)	δRMSE (mg/L)	δRMSE %
Congo	3.8	4.5	5.4	0.94	0.20	0.98	1.63							
ARIX								0.94	0.15	0.07	0.98	1.6	0.03	1.6
S275_295								1.14	0.32	-1.46	0.98	1.59	0.04	2.2
**SUVA**								-0.49	0.16	-0.02	1.00	0.66	0.32	59.7
Pan-Arctic								0.24	0.06		0.98	1.85	-0.22	-11.0
DOC_{LS}								603.9	124.8		1.00	2.32	-0.69	-42.3
DOC_{UV}								1507	32.2		1.00	23.5	-21.9	-1343.6
S. America	0.9	2.6	3.4	0.67	0.32	0.93	1.77							
**ARIX**								0.37	0.88	-1.02	0.95	1.39	0.38	21.3
**S275_295**								0.53	0.75	-5.78	0.94	1.71	0.24	12.2
**SUVA**									0.75	-0.13	0.99	0.70	1.07	60.4
Pan-Arctic								0.59	0.08		-0.12	3.52	-1.75	-99.3
DOC_{LS}								3156	-9.63		0.16	3.98	-2.20	-124
DOC_{UV}								1507	32.2		-0.04	5.87	-4.09	-230
Australia	1.7	3.8	5.3	0.58	0.20	0.94	0.51							
**ARIX**								0.56	0.23	-0.04	0.94	0.50	0.01	1.4
S275_295								0.58	0.19	0.05	0.94	0.51	0.00	-0.6
**SUVA**								0.17	0.48	-0.06	0.98	0.28	0.23	44.8
Pan-Arctic								1.05	0.03		0.91	0.70	-0.19	-38.0
DOC_{LS}								1746	-5.34		0.82	0.81	-0.30	-58.8
DOC_{UV}								1507	32.2		0.73	0.89	-0.38	-74.5
Horsens	2.1	3.5	4.5	1.48	0.24	0.92	0.94							
**ARIX**								0.60	0.47	-0.28	0.95	0.73	0.21	22.2
**S275_295**								1.44	0.33	-1.23	0.92	0.93	0.01	0.9
**SUVA**									0.63	-0.10	0.99	0.27	0.67	71.2
Pan-Arctic								1.21	0.03		0.90	1.39	-0.45	-48.2
DOC_{LS}								1423	130		0.86	1.12	-0.18	-19.1
DOC_{UV}								1507	32.2		0.87	1.43	-0.49	-52.1

Table S4 ctd.:

Model	SUVA quantiles 5%	50%	95%	DOC=$b_0 + b_1 \times A_{254}$ b_0	b_1	R^2	RMSE (mg/L)	ARINT or Pan-Arctic Model b_0	b_1	b_2	R^2	RMSE (mg/L)	δRMSE (mg/L)	δRMSE %
Everglades	1.0	2.4	3.8	5.96	0.19	0.83	1.75							
**ARIX**								2.86	0.97	-0.82	0.95	0.93	0.825	47.0
**S275_295**								1.85	1.04	-14.50	0.98	0.62	1.13	64.6
**SUVA**								2.69	0.60	-0.10	0.96	0.82	0.93	53.2
**Pan-Arctic**								0.47	0.07		0.93	1.27	0.48	27.6
**DOC_{LS}**								943	300		0.92	1.15	0.60	34.3
DOC_{UV}								1507	32.2		0.89	3.26	-1.51	-86.3
Yukon Lakes	1.1	2.5	3.6	5.90	0.33	0.52	7.23							
**ARIX**								3.00	1.01	-1.18	0.79	4.77	2.46	34.0
**S275_295**								3.36	1.01	-13.15	0.86	3.93	3.30	45.7
**SUVA**								2.10	0.86	-0.19	0.92	3.00	4.23	58.5
**Pan-Arctic**								0.71	0.06		0.92	2.93	4.30	59.5
**DOC_{LS}**								1820	-317		0.76	6.51	3.30	33.6
**DOC_{UV}**								1507	32.2		0.66	7.11	2.70	27.5
Alaska Rivers	2.4	3.6	4.0	0.45	0.25	1.00	0.33							
**ARIX**								0.38	0.30	-0.06	1.00	0.32	0.014	3.5
**S275_295**								0.38	0.37	-1.62	1.00	0.30	0.03	9.5
**SUVA**								0.15	0.54	-0.07	1.00	0.13	0.20	60.8
Pan-Arctic								0.55	0.07		1.00	0.45	-0.12	-35.7
DOC_{LS}								1911	6.32		0.99	0.68	-0.33	-94.3
DOC_{UV}								1507	32.2		0.99	2.11	-1.76	-503
SUEZ	0.6	2.0	4.2	1.51	0.21	0.81	1.06							
**ARIX**								0.64	0.50	-0.31	0.89	0.79	0.27	25.6
S275_295								*n.d.*	*n.d.*	*n.d.*	*n.d.*	*n.d.*	*n.d.*	*n.d.*
**SUVA**								0.43	0.56	-0.07	0.94	0.61	0.45	42.6
Pan-Arctic								*n.d.*	*n.d.*		*n.d.*	*n.d.*	*n.d.*	*n.d.*
DOC_{LS}								*n.d.*	*n.d.*		*n.d.*	*n.d.*	*n.d.*	*n.d.*
DOC_{UV}								*n.d.*	*n.d.*		*n.d.*	*n.d.*	*n.d.*	*n.d.*

Table S5: Model information criteria corresponding to Table S4. Each row shows values of Akaike's Information Criteria (AIC, AICc and CAIC) and the Bayesian Information Criterion (BIC) for the base model compared to an advanced model. Within a dataset, values for the base model vary between rows when units of predicted DOC differ (mgL^{-1} vs μM) or if sample size differs due to missing data (e.g. undefined spectral slopes). Values can only be compared for the same criterion within the same dataset and within a single row, or between rows only if both rows have identical values in the base model, since in this case the models are derived from identical raw data. Measured ARINT model parameters are A_{254} and ARIX/SUVA/S_{275_295}. Pan-Arctic Model parameters are a_{350} and S_{275_295}. Measured DOC_{LS} and DOC_{UV} parameters are a_{275}, a_{295}, a_{380}, and a_{443}. No models requiring spectral slopes were calculated for the SUEZ dataset due to incomplete absorbance data (*n.d.*). The best model in each pair of competing models is the one that minimises the values of most or all information criteria. Advanced models with significantly improved fit and improved predictions relative to the base model are highlighted in bold. ARINT models that include a non-significant interaction coefficient (b_2) are indicated by *.

				Base model: $DOC=b_0 + b_1 \times A_{254}$				Advanced model			
Dataset	Model	ARINT	n	AIC_0	AIC_{C0}	BIC_0	$CAIC_0$	AIC	AIC_C	BIC	CAIC
Congo	ARINT	*ARIX	131	504	498	500	502	499	491	493	496
Congo	ARINT	*S_{275_295}	131	504	498	500	502	498	490	492	495
Congo	**ARINT**	**SUVA**	131	504	498	500	502	-52	-60	-58	-55
Congo	PanArctic		131	504	498	500	502	555	549	551	553
Congo	DOC_{LS}		130	1650	1646	1644	1648	1742	1738	1736	1740
Congo	DOC_{UV}		130	1650	1646	1644	1648	2369	2369	2369	2369
S. America	**ARINT**	**ARIX**	106	425	419	421	423	376	368	370	373
S. America	**ARINT**	**S_{275_295}**	86	363	357	359	361	343	335	337	340
S. America	**ARINT**	**SUVA**	106	425	419	421	423	230	224	226	228
S. America	PanArctic		86	363	357	359	361	482	476	478	480
S. America	DOC_{LS}		104	1339	1335	1333	1337	1509	1505	1503	1507
S. America	DOC_{UV}		104	1339	1335	1333	1337	1585	1585	1585	1585
Australia	**ARINT**	**ARIX**	229	343	337	339	341	337	329	331	334
Australia	ARINT	*S_{275_295}	229	343	337	339	341	345	337	339	342
Australia	**ARINT**	**SUVA**	229	343	337	339	341	65	57	59	62
Australia	PanArctic		229	343	337	339	341	543	537	539	541
Australia	DOC_{LS}		228	2359	2355	2353	2357	2571	2567	2565	2569
Australia	DOC_{UV}		228	2359	2355	2353	2357	2609	2609	2609	2609
Horsens	**ARINT**	**ARIX**	318	885	879	881	883	736	728	730	733
Horsens	**ARINT**	**S_{275_295}**	318	885	879	881	883	875	867	869	872
Horsens	**ARINT**	**SUVA**	318	885	879	881	883	151	143	145	148
Horsens	PanArctic		318	885	879	881	883	1177	1171	1173	1175
Horsens	DOC_{LS}		307	3552	3548	3546	3550	3661	3657	3655	3659
Horsens	DOC_{UV}		307	3552	3548	3546	3550	3838	3838	3838	3838
Everglades	**ARINT**	**ARIX**	12	52	46	48	50	38	30	32	35
Everglades	**ARINT**	**S_{275_295}**	12	52	46	48	50	28	20	22	25
Everglades	**ARINT**	**SUVA**	12	52	46	48	50	35	27	29	32
Everglades	PanArctic		12	52	46	48	50	52	46	48	50
Everglades	**DOC_{LS}**		12	158	154	152	156	148	144	142	146

Everglades	DOC_{UV}		12	158	154	152	156	169	169	169	169
Alaska Rivers	**ARINT**	**ARIX**	53	37	31	33	35	35	27	29	32
Alaska Rivers	**ARINT**	**S_{275_295}**	53	37	31	33	35	30	22	24	27
Alaska Rivers	**ARINT**	**SUVA**	53	37	31	33	35	-58	-66	-64	-61
Alaska Rivers	PanArctic		51	36	30	32	34	74	68	70	72
Alaska Rivers	DOC_{LS}		45	435	431	429	433	495	491	489	493
Alaska Rivers	DOC_{UV}		45	435	431	429	433	599	599	599	599
Yukon Lakes	**ARINT**	**ARIX**	89	609	603	605	607	537	529	531	534
Yukon Lakes	**ARINT**	**S_{275_295}**	89	609	603	605	607	502	494	496	499
Yukon Lakes	**ARINT**	**SUVA**	89	609	603	605	607	454	446	448	451
Yukon Lakes	**PanArctic**		89	609	603	605	607	493	487	489	491
Yukon Lakes	**DOC_{LS}**		90	1466	1462	1460	1464	1393	1389	1387	1391
Yukon Lakes	**DOC_{UV}**		90	1466	1462	1460	1464	1405	1405	1405	1405
SUEZ	**ARINT**	**ARIX**	57	173	167	169	171	141	133	135	138
SUEZ	ARINT	S_{275_295}	0	*n.d.*	*n.d.*	*n.d.*	*n.d.*	*n.d.*	*n.d.*	*n.d.*	*n.d.*
SUEZ	**ARINT**	**SUVA**	57	173	167	169	171	112	104	106	109
SUEZ	PanArctic		0	*n.d.*	*n.d.*	*n.d.*	*n.d.*	*n.d.*	*n.d.*	*n.d.*	*n.d.*
SUEZ	DOC_{LS}		0	*n.d.*	*n.d.*	*n.d.*	*n.d.*	*n.d.*	*n.d.*	*n.d.*	*n.d.*
SUEZ	DOC_{UV}		0	*n.d.*	*n.d.*	*n.d.*	*n.d.*	*n.d.*	*n.d.*	*n.d.*	*n.d.*

Table S6: Percentiles (5% and 95%) for the values of P_π and b_2P_π/b_1 in ARINT models. Values are shown only in cases where the ARINT model significantly improved upon the base model.

			Dataset							
Pi	Variable	Percentile	*Congo*	*S. America*	*Australia*	*Horsens*	*Everglades*	*Alaska Rivers*	*Yukon Lakes*	*SUEZ*
SUVA	P_π	5	3.84	1.28	1.71	2.05	1.03	2.45	1.07	0.64
SUVA	P_π	95	5.42	5.14	5.26	4.55	3.82	4.03	3.63	4.19
ARIX	P_π	5		0.26	0.48	0.36	0.55	0.62	0.39	0.30
ARIX	P_π	95		0.66	1.02	0.78	0.95	0.87	0.68	0.82
$1/S_{275\_295}$	P_π	5		0.06		0.04	0.03	0.05	0.03	*n.d.*
$1/S_{275\_295}$	P_π	95		0.09		0.07	0.06	0.08	0.06	*n.d.*
SUVA	b_2P_π/b_1	5	-0.37	-0.21	-0.21	-0.35	-0.18	-0.33	-0.23	-0.09
SUVA	b_2P_π/b_1	95	-0.52	-0.85	-0.63	-0.61	-0.66	-0.54	-0.78	-0.56
ARIX	b_2P_π/b_1	5		-0.38	-0.09	-0.21	-0.47	-0.12	-0.46	-0.19
ARIX	b_2P_π/b_1	95		-0.76	-0.18	-0.47	-0.80	-0.17	-0.80	-0.52
$1/S_{275\_295}$	b_2P_π/b_1	5		-0.47		-0.22	-0.44	-0.21	-0.39	*n.d.*
$1/S_{275\_295}$	b_2P_π/b_1	95		-0.69		-0.28	-0.78	-0.35	-0.83	*n.d.*

References

1. S. E. Johnston *et al.*, Controls on Riverine Dissolved Organic Matter Composition Across an Arctic-Boreal Latitudinal Gradient. *J. Geophys. Res.-Biogeosci.* **126**, e2020JG005988 (2021).
2. S. E. Johnston *et al.*, Hydrologic connectivity determines dissolved organic matter biogeochemistry in northern high-latitude lakes. *Limnol Oceanogr* **65**, 1764-1780 (2020).
3. S. A. Timko, C. Romera-Castillo, R. Jaffé, W. J. Cooper, Photo-reactivity of natural dissolved organic matter from fresh to marine waters in the Florida Everglades, USA. *Environmental science. Processes & impacts* **16**, 866-878 (2014).
4. M. Philibert *et al.*, Drinking water aromaticity and treatability is predicted by dissolved organic matter fluorescence. *Wat Res* **220**, 118592 (2022).
5. C. A. Stedmon, S. Markager, Resolving the variability of dissolved organic matter fluorescence in a temperate estuary and its catchment using PARAFAC analysis. *Limnol Oceanogr* **50**, 686-697 (2005).
6. S. Acharya *et al.*, Relevance of tributary inflows for driving molecular composition of dissolved organic matter (DOM) in a regulated river system. *Wat Res* **237**, 119975 (2023).
7. T. Lambert, S. Bouillon, F. Darchambeau, P. Massicotte, A. V. Borges, Shift in the chemical composition of dissolved organic matter in the Congo River network. *Biogeosciences* **13**, 5405-5420 (2016).
8. D. Graeber, J. Gelbrecht, M. T. Pusch, C. Anlanger, D. von Schiller, Agriculture has changed the amount and composition of dissolved organic matter in Central European headwater streams. *Sci Tot Env* **438**, 435-446 (2012).
9. A. M. Kellerman *et al.*, Unifying Concepts Linking Dissolved Organic Matter Composition to Persistence in Aquatic Ecosystems. *Environ Sci Technol* **52**, 2538-2548 (2018).
10. R. Gonçalves-Araujo, M. A. Granskog, C. L. Osburn, P. Kowalczuk, C. A. Stedmon, A Pan-Arctic Algorithm to Estimate Dissolved Organic Carbon Concentrations From Colored Dissolved Organic Matter Spectral Absorption. *Geophys. Res. Lett.* **50**, e2023GL105028 (2023).
11. M. Yan, S. Mo, Z. Liu, G. Korshin, Absorptivity Inversely Proportional to Spectral Slope in CDOM. *Environ Sci Technol* **59**, 7156-7164 (2025).
